# PET imaging of P2X_7_R in the experimental autoimmune encephalomyelitis model of multiple sclerosis using [^11^C]SMW139

**DOI:** 10.1186/s12974-020-01962-7

**Published:** 2020-10-14

**Authors:** Wissam Beaino, Bieneke Janssen, Esther Kooijman, Ricardo Vos, Robert C. Schuit, James O’Brien-Brown, Michael Kassiou, Bert van het Hof, Danielle J. Vugts, Helga E. de Vries, Albert D. Windhorst

**Affiliations:** 1 Department of Radiology and Nuclear Medicine, Amsterdam UMC, location VUmc, Amsterdam, The Netherlands; 2 Present address: Department of Radiology, Perelman School of Medicine, University of Pennsylvania, Philadelphia, PA USA; 3 School of Chemistry, The University of Sydney, Sydney, NSW Australia; 4 Department of Molecular Cell Biology and Immunology, AUMC MS Center Amsterdam, Amsterdam Neuroscience, Amsterdam University Medical Center, Amsterdam, The Netherlands

**Keywords:** Microglia, Neuro-inflammation, PET imaging, P2X_7_R, Multiple sclerosis

## Abstract

**Background:**

Non-invasive imaging of the activation status of microglia and the ability to identify a pro- or anti-inflammatory environment can provide valuable insights not only into pathogenesis of neuro-inflammatory and neurodegenerative diseases but also the monitoring of the efficacy of immunomodulatory therapies. P2X_7_R is highly expressed on pro-inflammatory microglia and [^11^C]SMW139, a specific P2X_7_R tracer for positron emission tomography imaging, showed good pharmacokinetics, stability, and brain permeability in vivo. Our objective was to evaluate the potential of [^11^C]SMW139 for PET imaging of neuroinflammation in vivo in the experimental autoimmune encephalomyelitis (EAE) model.

**Methods:**

We induced EAE in Lewis rats by immunization with MBP 69-88 in complete Freund’s adjuvant (CFA). We determined the affinity of [^11^C]SMW139 to human and rat P2X_7_R using saturation binding assay. Using this tracer, PET imaging was performed at the peak of disease and in the recovery phase. In vivo blocking experiments were conducted to validate the specific brain uptake of the tracer. Immunohistochemistry staining and autoradiography were performed to evaluate the level of neuroinflammation and validate the specific binding of [^11^C]SMW139.

**Results:**

[^11^C]SMW139 showed good affinity for the rat P2X_7_R with a K_d_ of 20.6 ± 1.7 nM. The uptake of [^11^C]SMW139 was significantly higher in EAE animals at the peak of disease compared to the recovery phase but not in CFA control animals. The amplitude of increase of [^11^C]SMW139 uptake showed significant positive correlation with clinical scores mainly in the spinal cord (Pearson = 0.75, Spearman = 0.76; *p* < 0.0001). Treating EAE animals with P2X_7_R antagonist JNJ-47965567 blocked the uptake of [^11^C]SMW139 in the spinal cord, cerebellum, and brain stem, demonstrating specific accumulation of the tracer. P-glycoprotein blocking with tariquidar (30 mg/kg) did not affect tracer penetration in the brain showing that [^11^C]SMW139 is not a Pgp substrate.

**Conclusion:**

Our data shows that [^11^C]SMW139 is a promising PET tracer for imaging neuroinflammation and evaluating the dynamics of pro-inflammatory microglia in the brain. This can provide crucial insights into the role of microglia in disease progression and enables the development of novel treatment strategies aimed at modulating the immune response in order to promote neuroprotection.

## Introduction

Neuroinflammation is a common feature across many neurodegenerative diseases like multiple sclerosis (MS) and Alzheimer’s and Parkinson’s disease and is believed to contribute to the detrimental factors that lead to neurodegeneration. MS is an inflammatory autoimmune disease of the central nervous system (CNS) mainly associated with the development of demyelinating plaques and axonal degeneration [1]. Microglia are major players in disease pathogenesis and play a dual role in its progression (mediated by pro-inflammatory microglia) as well as in the resolution of the disease (through anti-inflammatory microglia) [2, 3]. However, the exact role of microglia and their activation status during the disease process remains poorly understood which is partially due to limited diagnostic tools to evaluate the dynamics of inflammation in the brain.

Positron emission tomography (PET) imaging provides unique capabilities for non-invasive visualization and quantification of molecular targets in the brain. To image the process of neuro-inflammation, translocator protein (TSPO) has been one of the most investigated targets for PET tracer development and imaging [4, 5]. However, the use of TSPO tracers is limited mainly due to the existing genetic polymorphism affecting the binding of TSPO tracers in humans, next to its intracellular localization and expression on endothelial cells, making it less than ideal target [6, 7]. In recent years, the P2X_7_ receptor (P2X_7_R) emerged as a new promising target for imaging neuroinflammation. P2X_7_R is an adenosine triphosphate-gated ion channel that belongs to the family of the purinergic receptors. P2X_7_R is expressed on cells from myeloid origin such as macrophages and microglia [8, 9] and is strongly connected to inflammatory processes where it plays an important role in the activation of the inflammasome and the release of interleukin (IL)-1β [10–15]. We recently showed that P2X_7_R is highly expressed in brain tissue from Lewis rats with experimental autoimmune encephalomyelitis (EAE), an animal model for MS, as well as in active and chronic active white matter lesions in post-mortem tissues of MS patients. Importantly, we demonstrated that expression of P2X_7_R on human microglia in vitro is highly upregulated under pro- but not anti-inflammatory conditions [16]. Few PET tracers targeting P2X_7_R have recently been reported that display good affinity and blood brain barrier (BBB) permeability. For instance, Berdyyeva et al. evaluated a fluor-18 labeled P2X_7_R antagonist, [^18^F]JNJ-64413739, in a neuro-inflammatory model using intracerebral lipopolysaccharide (LPS) injection and found an increase of tracer uptake in the LPS-injected part of the brain compared to the contralateral side [17]. The same tracer was also evaluated in non-human primates (NHP) and showed target engagement and dose-dependent occupancy in the brain [18]. Furthermore, Ory et al. evaluated [^11^C]JNJ54173717 for targeting and binding to P2X_7_R in vitro and in vivo in rats as well as in NHP and demonstrated good brain permeability and target engagement in the brain [19]. A recent first in human study investigated the biodistribution, dosimetry, kinetic modeling, and short-term test-retest variation of [^11^C]JNJ54173717 in healthy volunteers and PD patients and showed that the tracer is safe and suitable for quantifying P2X_7_R expression in human brain; however, no significant difference in P2X_7_R binding were found between both groups [20].

We previously reported [^11^C]SMW139 as a promising P2X_7_R PET tracer with the capacity to cross the BBB and engage the target in the brain in an AAV-hP2X_7_R model [21]. In this study, we assessed its potential for imaging neuroinflammation in the EAE model in Lewis rats. We evaluated the uptake of [^11^C]SMW139 at the peak of inflammation and compared it to the uptake in the recovery phase. We showed the specific binding of [^11^C]SMW139 to P2X_7_R in vivo by blocking with JNJ-47965567 [22], and we subsequently performed autoradiography and immunohistochemistry for ex vivo validation of tracer binding. We also investigated the correlation between tracer uptake and disease severity.

## Methods

### Reagents and PET tracer synthesis

Chemicals were obtained from commercial sources and used without further purification. Solvents were purchased from Sigma-Aldrich (Zwijndrecht, the Netherlands), Merck (Darmstadt, Germany), and Biosolve (Valkenswaard, the Netherlands) and used as received unless stated otherwise. Tetrahydrofuran (THF) was first distilled from LiAlH_4_ and then stored on 3 Å molecular sieves. [^3^H]methyl nosylate stock solution (717 MBq/mL in toluene, molar activity of 3.08 GBq μmol^− 1^) was obtained from Novandi Chemistry AB (Södertälje, Sweden) and dried at 60 °C under an argon flow prior to use. [^11^C]SMW139 was synthesized in a similar manner as previously described [21]. [^3^H]SMW139 was synthesized in a slightly adapted manner using [^3^H]methyl nosylate and a longer reaction time (16 h) at room temperature (RT). Radiopharmaceutical nomenclature was applied as in Coenen et al. [23].

### Cell transfection and membrane preparation

HEK293 cells were purchased from Sigma-Aldrich (Zwijndrecht, the Netherlands) and cultured in Dulbecco’s modified Eagle’s medium supplemented with 10% fetal bovine serum (FBS), penicillin (100 units/mL), streptomycin (100 μg/mL), and L-glutamine (300 μg/mL) and maintained at 37 °C, 5% CO_2_.

Stable transfection of HEK293 cells with human P2X_7_R (hP2X_7_R) or rat P2X_7_R (rP2X_7_R) was done using lipofectamine 2000 (Invitrogen, San Diego, CA). Plasmid pcDNA3.1(+)-hP2X_7_R-P2A-eGFP and pcDNA3.1(+)-rP2X_7_R-P2A-eGFP were purchased from Genscript (Tokyo, Japan). Briefly, cells were seeded in 24-well plates overnight to reach 70% confluency and were then transfected with the plasmid (0.8 μg/well) using lipofectamine 2000 according to the manufacturer’s protocol. After 24 h of transfection, cells were transferred to a 6-well plate and kept to recover for 24 h; G-418 (1 mg/mL) (Sigma-Aldrich Zwijndrecht, the Netherlands) was added for positive transfection selection. Media with G-418 was refreshed every other day for 10 days, and cells were sorted for eGFP positivity on a BD FACSARIA Fusion (BD Bioscience, Vianen, The Netherlands). The sorting enrichment of P2X_7_R expressing cells was > 95%.

For membrane preparation of HEK293, P2X_7_R-expressing cells were collected using a scraper and washed twice with PBS. Cells were re-suspended in Tris/HCl buffer (50 mM, pH 7.4) containing NaCl (140 mM), EDTA (2 mM), and protease inhibitors (cOmplete™ ULTRA Tablets, Sigma-Aldrich) at 2 × 10^6^ cell/mL. Membranes were then broken using a probe sonicator (2 × 15 s at full speed) on ice. The homogenate was centrifuged at 20,000 round per minute (RPM) for 30 min at 4 °C. The supernatant was discarded and the pellet re-suspended in Tris/HCl buffer (50 mM, pH 7.4). Protein content was determined using BCA assay (BCA Protein Assay Kit, Pierce), and membranes were aliquoted and stored at − 80 °C.

### Saturation binding assay

Cell membranes (5 μg) were mixed with an increased concentration of [^3^H]SMW139 (0.5, 1, 2, 3, 5, 7, 10, 15, 20, 40, 60, 80 nM) in binding buffer (50 mM Tris-HCl pH 7.4, 120 mM NaCl, 5 mM KCl, 5 mM MgCl_2_, 1 mM EDTA) (500 μL) and shaken for 2 h at room temperature (RT). Blocking was also performed to determine the non-specific binding. Membranes were incubated with an increased concentration of [^3^H]SMW139 and blocker JNJ-54173717 (10 μM) for 2 h at RT with shaking. Membranes were harvested on polyethylamine-treated fiber glass sheets using a Brandel harvesting system (Gaithersburg, MD, USA) and were quickly washed with cold PBS and collected into tubes. Scintillation liquid (Optiphase HISAFE 3, Perkin Elmer) was added. The radioactivity in each sample was measured using a scintillation beta counter (Hidex 300 SL). The data analysis and the binding affinity K_d_ was determined using Graphpad prism 5 (San Diego, CA, USA).

### Experimental autoimmune encephalomyelitis (EAE) model

Experimental autoimmune encephalomyelitis was induced in 11–15 weeks old female Lewis rats/*SsNHsd*, (Charles River, UK). Rats were immunized s.c. at two locations at the base of the back with an emulsion (200 μL) containing 200 μg synthetic guinea pig myelin basic protein peptide (gpMBP 69-88), 500 μg *Mycobacterium tuberculosis* type 37HRa (Difco, Detroit, MI, USA), 100 μL complete Freund’s adjuvant (CFA) (Difco), and 100 μL sterile water. Pertussis toxin (400 ng) (*Gibco*) was injected i.p. at day 0 and day 2 post-immunization. Rats were examined daily and clinical scores were graded on a scale from 1 to 5 for neurological signs. Severity of paresis was graded as follows: 0, clinically normal; 0.5, distal limp tail; 1, decreased tail tone or weak tail only; 1.5, limp tail and slight weakness in one of the hind limbs; 2, weak hind limbs; 2.5, one paralyzed hind limbs with instability in the other; 3, both hind limb paralysis; 3.5, both hind limb paralysis and weakness in one forelimb; 4, paralysis of hind and weakness of forelimbs; 5, paralysis of all limbs (quadriplegia). Animals were housed under standard laboratory conditions with water and food ad libitum. Animals immunized with CFA (200 μL) supplemented with *Mycobacterium tuberculosis* type 37HRa (6 mg/mL) were used as a control. Animal experiments were performed in accordance with the European Community Council Directive (2010/63/EU) for laboratory animal care and the Dutch Law on animal experimentation. The experimental protocol was validated and approved by the central committee for animal experimentation (CCD) and the local committee on animal experimentation of the VU University Medical Center.

### Metabolite study

Lewis rats/*SsNHsd* with EAE at the peak of the clinical symptoms were used in this experiment. Rats were injected with the tracer [^11^C]SMW139 (25–35 MBq; 0.2–0.7 nmol; *n* = 8, molar activity 103–122 GBq/μmol) via the tail vein catheter under isoflurane anesthesia (2–2.5% in 1 L/min). Rats were sacrificed at 15 or 45 min post-tracer injection (*n* = 4 per time point). Blood was collected via heart puncture into heparin tubes (DB Vacutainer, LH, Becton Dickinson, Franklin Lakes, NY, USA) and centrifuged at 4000 RPM for 5 min at 4 °C (Hettich Universal 32, Andreas Hettich GmbH & Co. KG, Tuttlingen, Germany). Plasma supernatant was separated from blood cells and loaded onto a C18 Sep-Pak (Waters, Milford, MA, USA), followed by washing with 3 mL of water to obtain the polar fraction. The non-polar fraction was then eluted with 2 mL of methanol and 1 mL of water and further analyzed by HPLC. The brain and spinal cord were put separately in a falcon tube containing 4 mL MeCN/H_2_O (50:50 v/v) and homogenized with a disperser (IKA T18 B Ultra-Turrax, IKA®-Werke GmbH & Co.KG, Staufen, Germany) before centrifugation (5 min, 4000 rpm, 20 °C, Hettich Universal 32). Supernatant was separated from brain precipitate and analyzed by HPLC. Analytical HPLC was performed with Dionex (Sunnyvale, CA, USA) UltiMate 3000 HPLC equipment with Chromeleon software (version 6.8) on a Gemini C18 5-μm (10 × 250 mm) column (Phenomenex, Torrance, CA, USA) with gradient and a mixture of acetonitrile (A) and 0.1% DIPA in water (B) as eluent according to the following scheme: 0 min, 60% B at 0.25 mL min^− 1^; 0.5 min, 60% B at 4 mL min^− 1^; 5.0 min, 10% B at 4 mL min^− 1^; 12.0 min, 10% B at 4 mL min^− 1^; 13.0 min, 60% B at 4.0 mL min^− 1^; 14.5 min, 60% B at 4 mL min^− 1^; and 15 min, 60% B at 0.25 mL min^− 1^. All separate fractions were counted for radioactivity in a Wizard Gamma counter 1470 or 2480 (Wallac/PerkinElmer, Waltham, MA, USA).

### Efflux pump blocking in vivo

Male Wistar rats (180–200 g) (*n* = 12) were purchased from Charles Rivers (Germany) and housed in groups with access to food and water ad libitum. For biodistribution study, rats were intravenously injected through a tail catheter with either [^11^C]SMW139 only (*n* = 4) (25–40 MBq, 0.2–0.9 nmol; molar activity 72–136 GBq/μmol) or tariquidar (15 mg/kg) formulated in dextrose solution 30 min prior injection of [^11^C]SMW139 (*n* = 4) (25–40 MBq, 0.2–0.9 nmol; molar activity 72–136 GBq/μmol). Animals were then sacrificed and the blood and brain were collected, weighed, and measured for radioactivity in a Perkin Elmer Wizard II gamma counter. Percent of injected dose per gram tissue (%ID/g) was calculated for blood and brain. For PET imaging, the same animals were used for baseline scan and tariquidar blocking scans (*n* = 4). For the baseline scan, rats were injected with [^11^C]SMW139 through a tail vein catheter and dynamic PET imaging was acquired for 45 minutes. Three hours later the block scan with tariquidar was performed. Tariquidar was injected intravenously through the tail vein catheter 30 min prior the injection of the [^11^C]SMW139 and the PET imaging acquisition. Dynamic PET imaging was performed with a Mediso nanoscan PET/CT and PET/MR (Mediso Ltd., Budapest, Hungary) equipped with identical PET components. PET scans were acquired in list mode and rebinned into the following frame sequence: 4 × 5, 4 × 10, 2 × 30, 3 × 60, 2 × 300, 1 × 600, 1 × 900, and 1 × 1200 s. Reconstruction was performed using a fully three-dimensional reconstruction algorithm (Tera-TomoTM, Mediso Ltd.) with 4 iterations and 6 subsets and an isotropic 0.4 mm voxel dimension. Images were analyzed using the VivoQuant software (Invicro, Boston, USA).

### PET imaging in the EAE model

PET imaging was performed with [^11^C]SMW139 in EAE rats at the maximum of the clinical signs and after full recovery from the clinical symptoms. Dynamic acquisition was performed for 45 min post-tracer injection. Regions-of-interest (ROI) were drawn manually on the spinal cord and by applying a brain atlas for the different brain regions (Additional file 1, Figure 1). PET imaging analysis was focused on the spinal cord, cerebellum, and brain stem as these are the main areas with the most prominent neuroinflammation in this model. Dynamic PET imaging was performed using small animal NanoPET/CT and NanoPET/MR scanners (Mediso Ltd., Budapest, Hungary) equipped with identical PET components. EAE rats (*n* = 23) and CFA rats (*n* = 4) were anaesthetized with 2–4% isoflurane in oxygen (1 L/min). Rats were positioned on the scanner bed and their respiratory rate was monitored during the entire scan, and anesthesia was adjusted whenever required. Dynamic PET scans were acquired immediately after intravenous (i.v.) administration of [^11^C]SMW139 (20–30 MBq, 0.2–0.7 nmol; molar activity 75–151 GBq/μmol) via a tail vein catheter. For blocking experiments, rats were injected subcutaneously with JNJ-47965567 (30 mg kg^− 1^) in 30% (2-hydroxypropyl)-β-cyclodextrin in water for injection 30 min prior to tracer injection. PET scans were acquired in list mode and rebinned into the following frame sequence: 4 × 5, 4 × 10, 2 × 30, 3 × 60, 2 × 300, 1 × 600, 1 × 900, and 1 × 1200 s. Reconstruction was performed using a fully three-dimensional reconstruction algorithm (Tera-TomoTM, Mediso Ltd.) with 4 iterations and 6 subsets, and an isotropic 0.4 mm voxel dimension. For gadolinium-enhanced MRI post-PET imaging acquisition, rats were injected with Dotarem (2 mL/kg, 1 mmol/kg) and 2D T1 scans were acquired using a T1 spin echo 2D sequence (T1SE2D) in the sagittal and coronal plans as preset in the Mediso nanoscan PET/MR scanner. Images were analyzed and quantified using the VivoQuant software (Invicro, Boston, USA), and region of interest (ROI) were applied using VivoQuant-integrated brain atlas fitting CT and MRI scans. Data were analyzed using Graphpad prism 5 (San Diego, CA, USA).

### In vivo blocking in the EAE rats

In vivo blocking studies were performed using a P2X_7_R antagonist JNJ-47965567 (30 mg/kg). Rats with severe EAE clinical signs (*n* = 6) were imaged twice a day or on consecutive days with and without blocker at the peak of the disease and in the recovery phase. The time difference between the first PET scan and the block scan was between 4 and 18 h. PET scans were analyzed and regions of interest for the spinal cord, cerebellum, and brain stem were normalized to a reference region in the forebrain. The forebrain was used as a reference region as no microglia activation or macrophage infiltration was observed in IHC and no difference in uptake of [^11^C]SMW139 was observed between scans at the peak of the disease and at the recovery phase in that region (Additional file 1, Figure 4).

### Autoradiography

For autoradiography, spinal cord tissues from EAE animals at the peak of the disease (*n* = 3) were used. Tissue cryosections (20 μm) were mounted on glass slides, air-dried, and stored at − 80 °C until use. Sections were washed three times for 5 min with assay buffer (50 mM Tris-HCl, pH 7.4) and then dried under an air flow. Sections were incubated with [^3^H]SMW139 (60 nM) only or with [^3^H] SMW139 (60 nM) together with JNJ-54173717 (100 μM) as blocker for 2 h at room temperature. Sections were washed 3 × 3 min with Tris-HCl (50 mM, pH 7.4) and dipped twice in deionized water. Sections were dried under an air flow and exposed to phosphor screen BAS-IP TR 2025 (General Electric, Eindhoven, the Netherlands) for 1 week. Phosphor screens were imaged using Typhoon FLA 7000 imager (General Electric, Eindhoven, the Netherlands). The intensity of the signal was quantified using image Quant software (General Electric, Eindhoven, the Netherlands).

### Immunohistochemistry

For immunohistochemistry, spinal cord tissue from animals in the EAE phase (severe and mild EAE), recovery phase, and CFA control were stained for IBA-1 and CD68 in order to evaluate the level of neuroinflammation. Paraffin-embedded spinal cord sections were de-paraffined with multiple xylene and ethanol bathes and then rehydrated in PBS. Antigen retrieval was performed in citrate buffer (0.01 M, pH 6) for 15 min at 90 °C. Sections were washed with PBS and incubated in 0.5% H_2_O_2_ in PBS for blocking endogenous peroxidase for 5 min at RT. Sections were washed three times for 5 min with PBS and blocked with super block (BSA 1%, normal goat serum 5%, cold fish skin gelatin 0.1%, Triton X-100 0.5%) or 2 % BSA for 30 min at RT followed by incubation with anti-CD68 (ED1) (1/100) (Hycult Biotech; HM3029) antibody in PBS/1% BSA for 1 h at RT. Subsequently, the slides were washed three times for 5 min with PBS and incubated with Dako envision kit for 1 h at RT and followed by washing three times for 5 min with PBS, and DAB solution (DAKO, Agilent technology, Middelburg, the Netherlands) was added till the desired intensity is obtained. Slides were counter-stained with hematoxylin and mounted with coverslips using Eukitt mounting media. For Rabbit anti-IBA-1 (1/1000) (Wako, 019-19741) staining, the same protocol was used but without the antigen retrieval step. Immunofluorescence double staining for P2X_7_R, CD11b and CD68 was performed as described in [16].

## Results

### Binding affinity of SMW139 to P2X_7_R

**Fig. 1:**
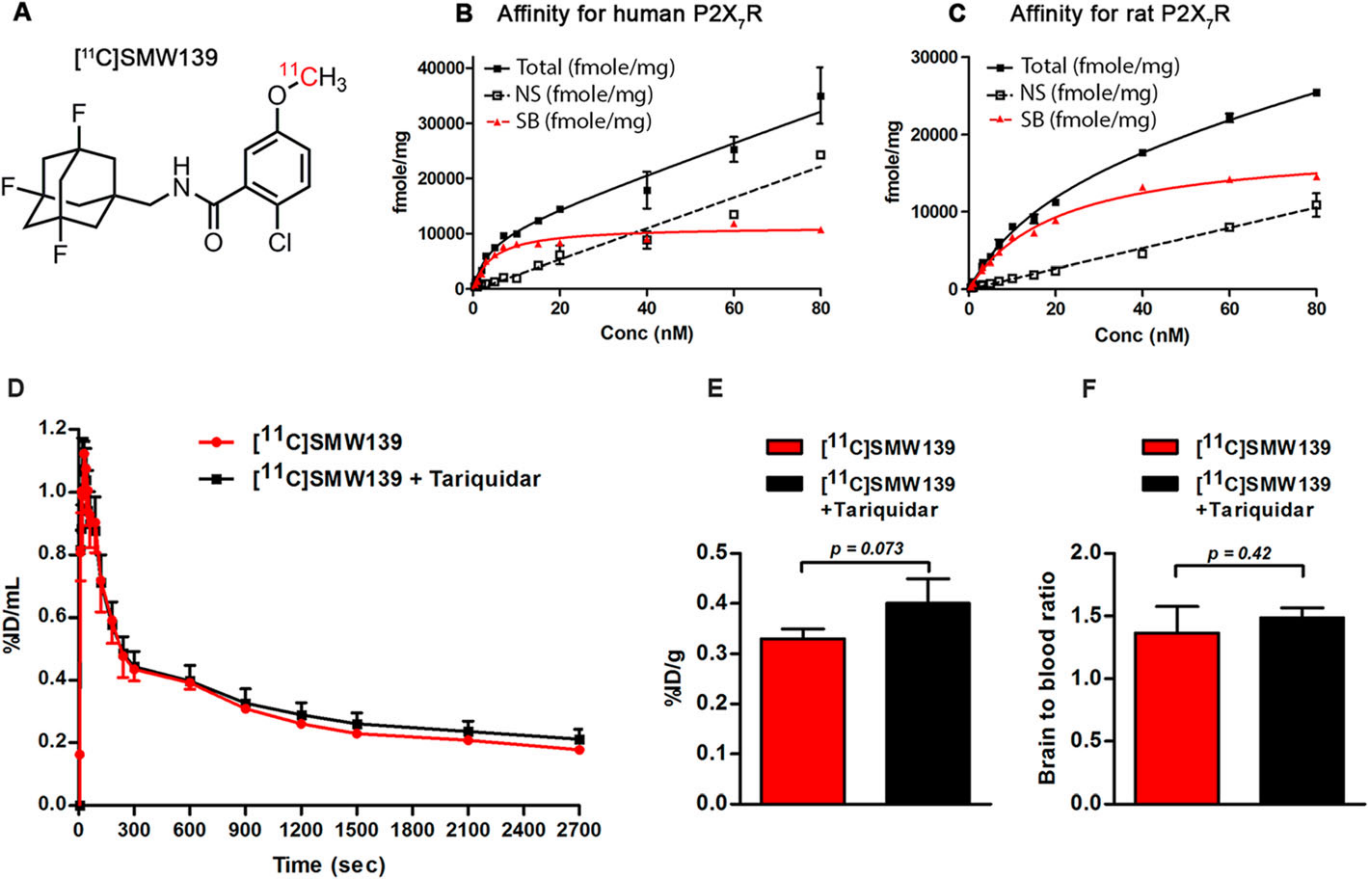
Chemical structure of [^11^C]SMW139 (**a**). The binding of [^3^H]SMW139 was determined by saturation binding assay to human or rat P2X_7_R (*n* = 3). Binding curves of [^3^H]SMW139 to human; binding affinity K_d_ = 4.6 ± 0.8 nM (**b**). Binding curves of [^3^H]SMW139 to rat P2X_7_R; binding affinity K_d_ = 20.6 ± 1.7 nM (**c**). In vivo P-glycoprotein blocking with tariquidar. Time activity curve showing the %ID/mL of the baseline scan with [^11^C]SMW139 in normal wild-type rats (*n* = 4) (red circle) and after blocking with tariquidar 15 mg/kg (*n* = 4) (black square) (**d**). Biodistribution showing the %ID/g (**e**) and the brain-to-blood ratio (**f**) of [^11^C]SMW139 in the brain of normal wild-type rats and after blocking with tariquidar (15 mg/kg). *p* value was determined by two-tailed *t* test. SB, specific binding, NS non-specific binding

SMW139 (Fig. 1a) is an allosteric antagonist developed for human P2X_7_R [24]. We performed a saturation binding assay in order to determine if the binding affinity for rat P2X_7_R is in the acceptable range for a PET tracer. The binding affinity (K_d_) for the rat P2X_7_R was fivefold lower compared to the affinity for human P2X_7_R (20.6 ± 1.7 nM and 4.6 ± 0.8 nM, respectively) (Fig. 1b, c).

### In vivo efflux pump blocking

P-glycoprotein (Pgp) is the main efflux pump at the BBB potentially limiting tracer uptake. In order to determine if [^11^C]SMW139 is a Pgp substrate, we performed a biodistribution and imaging study in normal Wistar rats with and without injection of the Pgp inhibitor tariquidar. Quantification of PET imaging showed identical time activity curves for [^11^C]SMW139 in the brain for both scans. The biodistribution study showed that brain uptake of [^11^C]SMW139 was comparable with and without tariquidar (≈ 0.4 %ID/g) (Fig. 1d–f).

### EAE model and study design

**Fig. 2:**
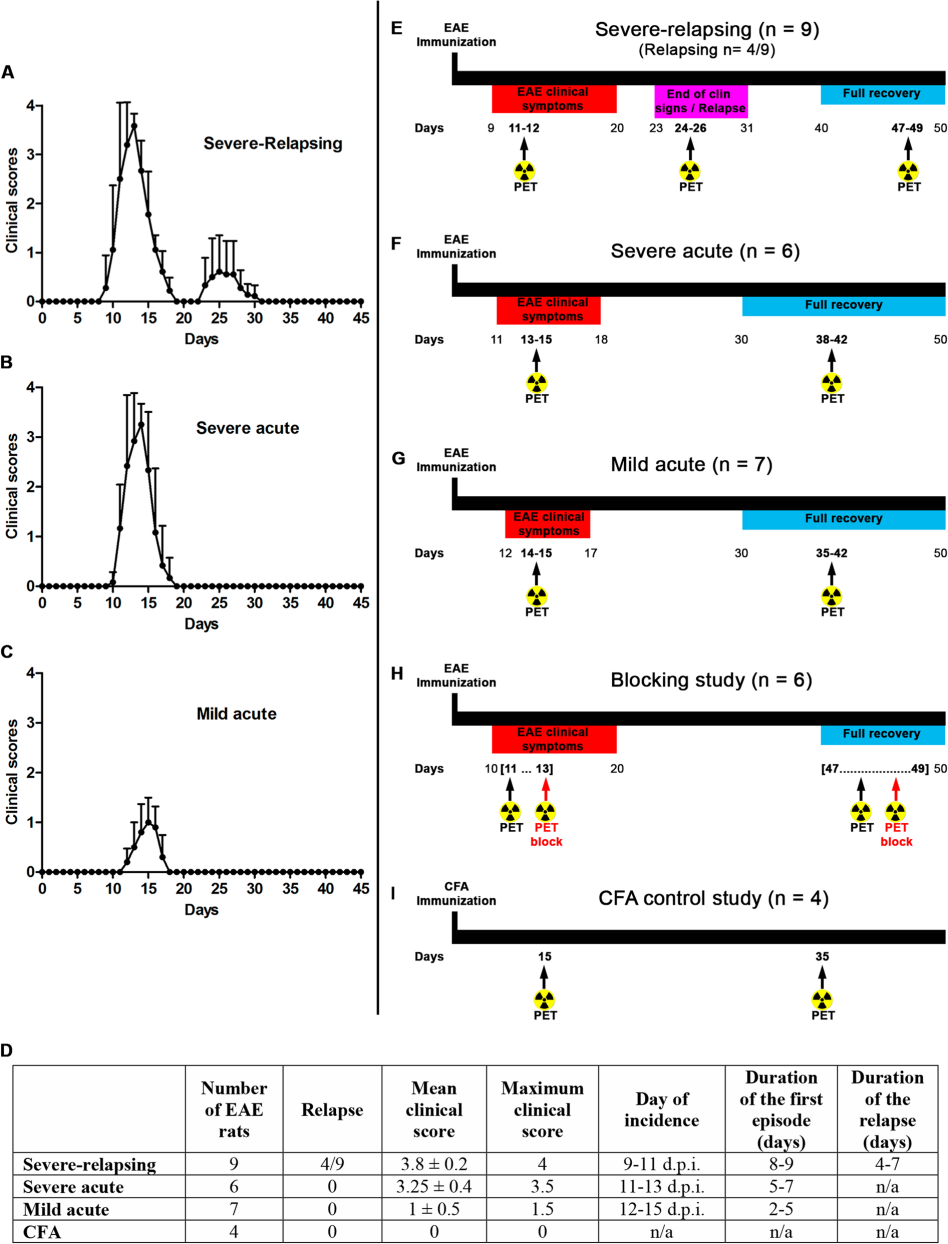
Clinical scores of EAE animals that developed severe-relapsing (*n* = 9) (**a**), severe acute (*n* = 6) (**b**), and mild acute (*n* = 7) (**c**) EAE. Table summarizing the information for the different EAE groups (**d**). Experimental planning of PET imaging in different EAE groups (severe-relapsing (**e**), severe acute (**f**), and mild acute (**g**)), blocking group (**h**), and control CFA group (**i**). Arrows are showing when PET imaging was performed

The severity of the EAE developed in the Lewis rats was heterogeneous, and four of the animals had an unexpected relapse after the initial EAE phase. Before the PET imaging analysis, rats were divided into three groups based on the day of incidence, severity of the clinical signs, the length of the first clinical period, and the presence of relapses. Seven rats developed mild EAE, six rats developed severe acute EAE, and nine rats developed severe-relapsing EAE including four relapsing rats. One EAE rat did not develop any clinical signs (Fig. 2a–d). One group immunized with CFA (*n* = 4) was used as control for PET imaging. All EAE rats were imaged at the peak of the EAE (maximum of the clinical signs) and at the recovery phase. Five rats of the severe-relapsing group were additionally imaged during the relapsing phase (days 24–26 post-immunization) including 2/5 that relapsed and 3/5 with no relapse. The CFA control group was imaged at the same time post-immunization as the EAE rats for comparison. The intervention overview for the different groups is summarized in Fig. 2e–i.

### [^11^C]SMW139 stability and metabolites in the EAE rats

[^11^C]SMW139 showed good plasma stability with 73 ± 7% and 37 ± 10% of intact tracer remaining at 15 and 45 min, respectively. [^11^C]SMW139 was more stable in the brain and spinal cord with 89 ± 5% and 89 ± 4% of intact tracer at 15 min respectively, and 66 ± 11% and 59 ± 8% at 45 min respectively.

### PET imaging of neuroinflammation in the EAE rats

**Fig. 3:**
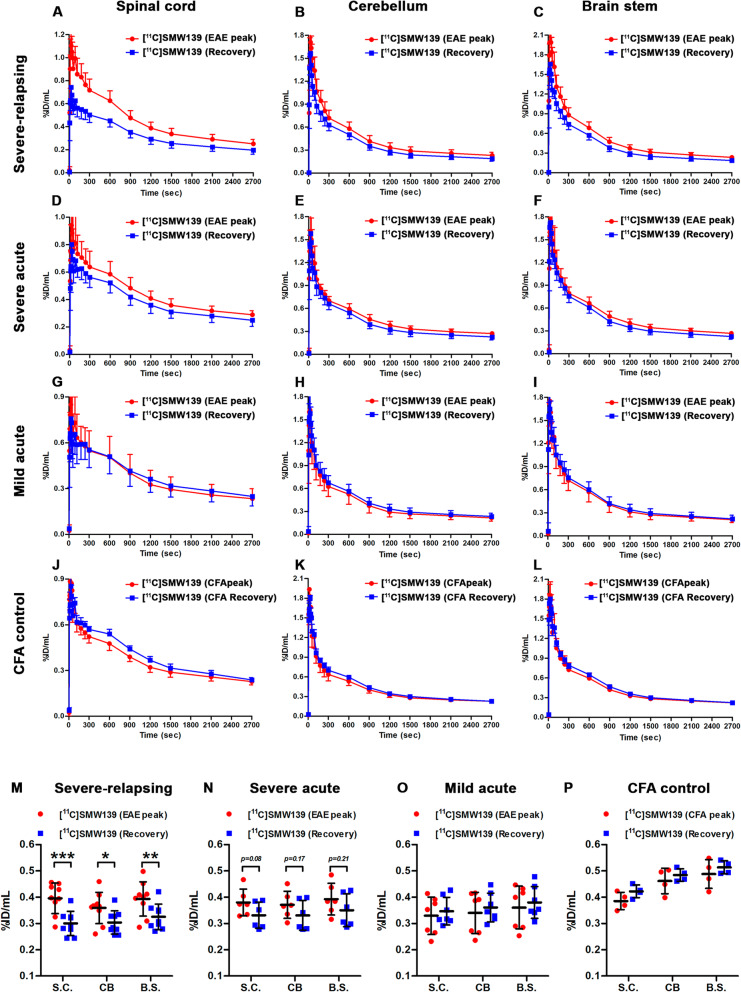
Time activity curve (TAC) of [^11^C]SMW139 uptake in the spinal cord, cerebellum, and brain stem (%ID/mL) at the peak of the EAE disease (red circle) and at the recovery phase (blue square). TAC of [^11^C]SMW139 in the severe-relapsing (**a**–**c**), severe acute (**d**–**f**), and mild acute (**g**–**i**) EAE animals and CFA control animals (**j**–**l**). Quantification of PET imaging in the spinal cord, cerebellum, and brain stem of a single-frame static reconstruction between 5 and 45 min post [^11^C]SMW139 injection in the severe-relapsing (*n* = 9) (**m**), severe acute (*n* = 6) (**n**), mild acute (*n* = 7) (**o**) EAE, and CFA control (*n* = 4) (**p**). S.C. spinal cord, CB cerebellum, B.S. brain stem. (*, *p* < 0.05; **, *p* < 0.001; ***, *p* < 0.0001). Data are expressed as percent injected dose per milliliter (%ID/mL).

**Fig. 4:**
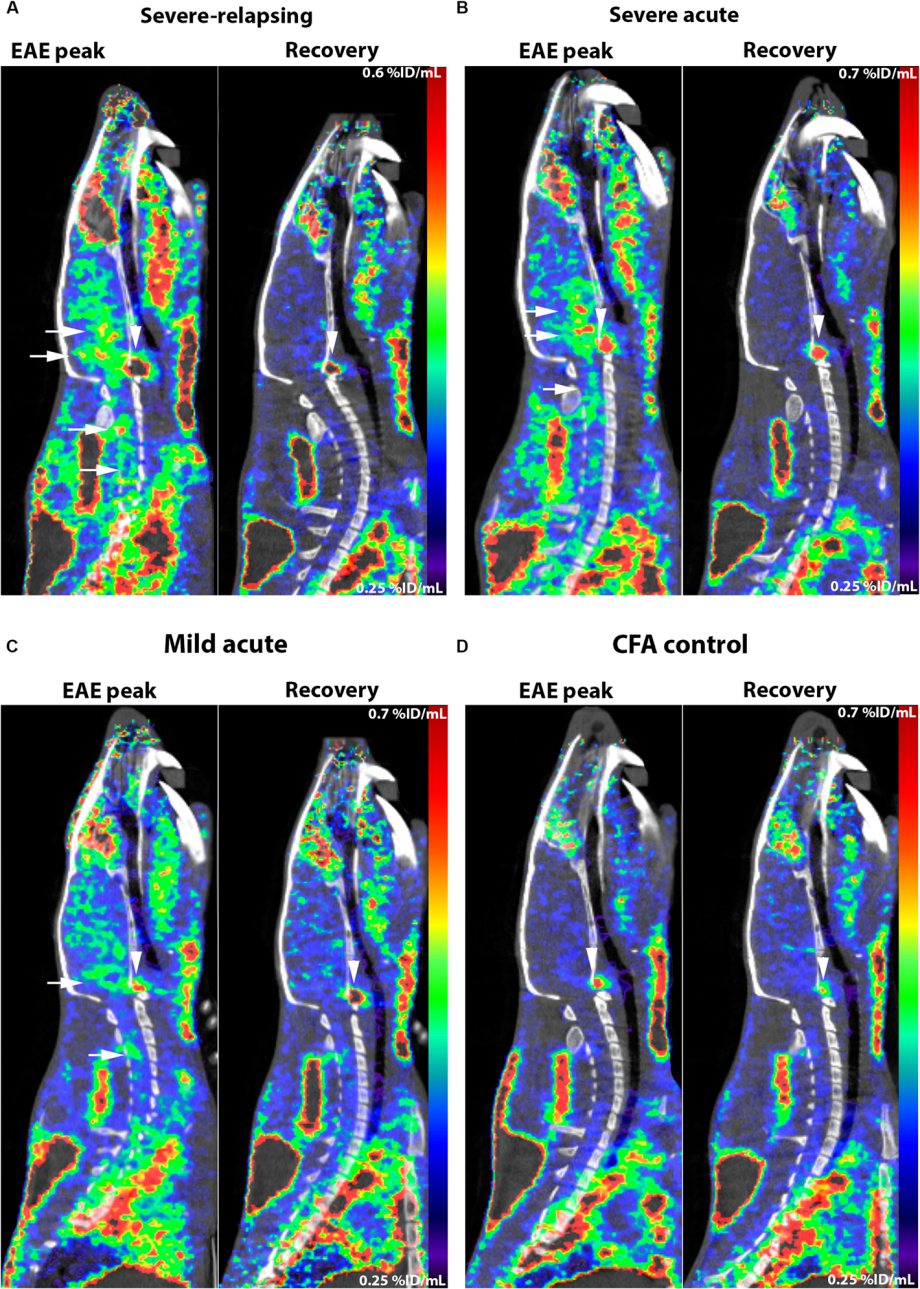
PET images of [^11^C]SMW139 in the EAE rats at the peak of the disease and in the recovery phase. Sagittal PET images extracted from the static reconstruction of the 5–45 min frame and showing [^11^C]SMW139 uptake in the brain and spinal cord (arrows) of severe-relapsing (**a**), severe acute (**b**), mild acute (**c**) EAE, and CFA control (**d**). Arrow heads are showing [^11^C]SMW139 uptake in a brain draining lymph node

For the severe-relapsing group, uptake of [^11^C]SMW139 in the spinal cord was significantly higher (37% by comparing the area under the curve (AUC) of the group) at the peak of disease compared to the recovery phase (Fig. 3a). In the severe acute EAE rats, the uptake of [^11^C]SMW139 in the spinal cord at the peak of the disease was higher compared to the recovery phase (AUC of the group 15% higher), but the difference was lower compared to the severe-relapsing group (Fig. 3d). In the mild acute EAE rats, we did not observe any difference in tracer uptake in the spinal cord between the disease and recovery phase (Fig. 3g) (Additional file 1, Figure 2). In the cerebellum and brain stem, uptake of [^11^C]SMW139 was also higher at the peak of the EAE compared to the recovery phase for the severe-relapsing (AUC of the group for the cerebellum 18% and brain stem 23% higher) (Fig. 3b, c) and the severe acute EAE (AUC of the group for the cerebellum 12% and brain stem 10% higher) (Fig. 3e, f), but not for the mild EAE group and CFA control (Fig. 3h–l). We also performed static reconstruction for the 5–45 min PET acquisition frame. For the severe-relapsing EAE rats, the uptake of [^11^C]SMW139 at the peak of the disease was significantly higher compared to the recovery phase in the spinal cord (0.4 ± 0.06 vs 0.3 ± 0.05 %ID/mL, respectively; *p* < 0.0001), in the cerebellum (0.36 ± 0.07 vs 0.3 ± 0.04 %ID/mL, respectively; *p* < 0.05), and in the brain stem (0.4 ± 0.07 vs 0.32 ± 0.05 %ID/mL, respectively; *p* < 0.001) (Fig. 3m). In the severe acute EAE rats, the uptake was also higher at the peak of the disease compared to the recovery phase in the spinal cord (0.38 ± 0.05 vs 0.33 ± 0.05% ID/mL, respectively; *p* = 0.08), cerebellum (0.37 ± 0.05 vs 0.33 ± 0.06 %ID/mL, respectively; *p* = 0.17), and brain stem (0.39 ± 0.06 vs 0.35 ± 0.06 %ID/mL, respectively; *p* = 0.21) (Fig. 3n). There was no significant different between the two scanning time points for the mild EAE rats (Fig. 3o). No significant difference was observed in the CFA control group (Fig. 3p). Representative PET images of sagittal section for the different EAE groups and CFA control at the peak of the disease and the recovery phase is shown in Fig. 4 (Additional file 1, Figures 7 and 9).

**Fig. 5:**
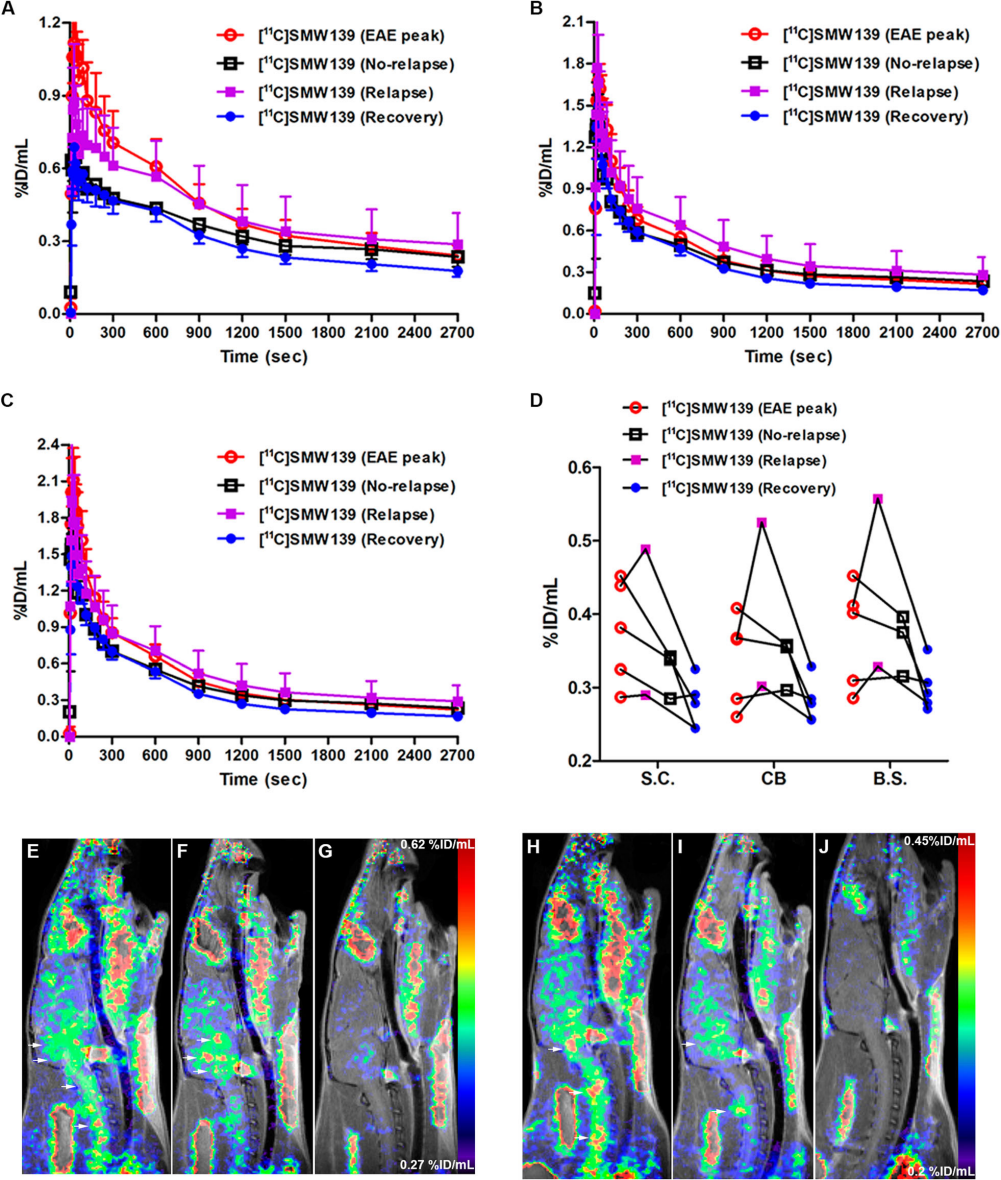
Time activity curves (TAC) of [^11^C]SMW139 uptake in the spinal cord (**a**), cerebellum (**b**), and brain stem (**c**) (%ID/mL) of five rats that developed severe EAE and of which two rats developed a relapse and three did not develop any relapse. TAC of all the rats at the peak of the disease (11–12 days post immunization (dpi)) (white circle), the two rats that developed a relapse during the relapse phase (24–26 dpi) (pink square), the three rats that did not develop a relapse but imaged at the same time as the relapse phase (24–26 dpi) (white square), and all the animals at the recovery phase (47–49 dpi) (blue circle). Quantification of PET imaging in the spinal cord, cerebellum, and brain stem of a single frame static reconstruction between 5 and 45 min post [^11^C]SMW139 injection (**d**). Sagittal PET images extracted from the static reconstruction of the 5–45 min frame and showing [^11^C]SMW139 uptake in the brain and spinal cord (arrows) of relapsing EAE rat (peak of the disease (**e**), relapse (**f**), recovery (**g)**) and non-relapsing rat (peak of the disease (**h**), no-relapse (**i**), recovery (**j**)). S.C. spinal cord, CB cerebellum, B.S. brain stem. Data are expressed as percent injected dose per milliliter (%ID/mL)

Four of the animals that developed severe EAE had an unexpected relapse after the initial EAE phase. Two of these animals were additionally imaged with [^11^C]SMW139 during the relapse together with three rats that had severe EAE without relapse in order to evaluate a differential uptake of [^11^C]SMW139 tracer between relapsing and non-relapsing animals. The tracer uptake in the severe-relapsing animals during the relapse phase did not significantly increase in the spinal cord but was increased in the cerebellum and brain stem compared to the peak of the disease. In the animal that did not show a relapse, the tracer uptake was decreased in the spinal cord, cerebellum, and brain stem (Fig. 5a–d). PET images in the relapsed animals showed several new focal spots with increased uptake in the cerebellum at the relapse phase that were not visible in the previous scan at the peak of the EAE which suggest the presence of new inflammatory lesion (Fig. 5e–j).

It is interesting to mention that the animal that did not develop EAE and imaged with [^11^C]SMW139 at the same time points as the other EAE animals did not show any significant difference of uptake at the peak of the disease compared to the recovery phase (Additional file 1, Fig. 3).

### Blocking of [^11^C]SMW139 uptake in vivo in the EAE rats

**Fig. 6:**
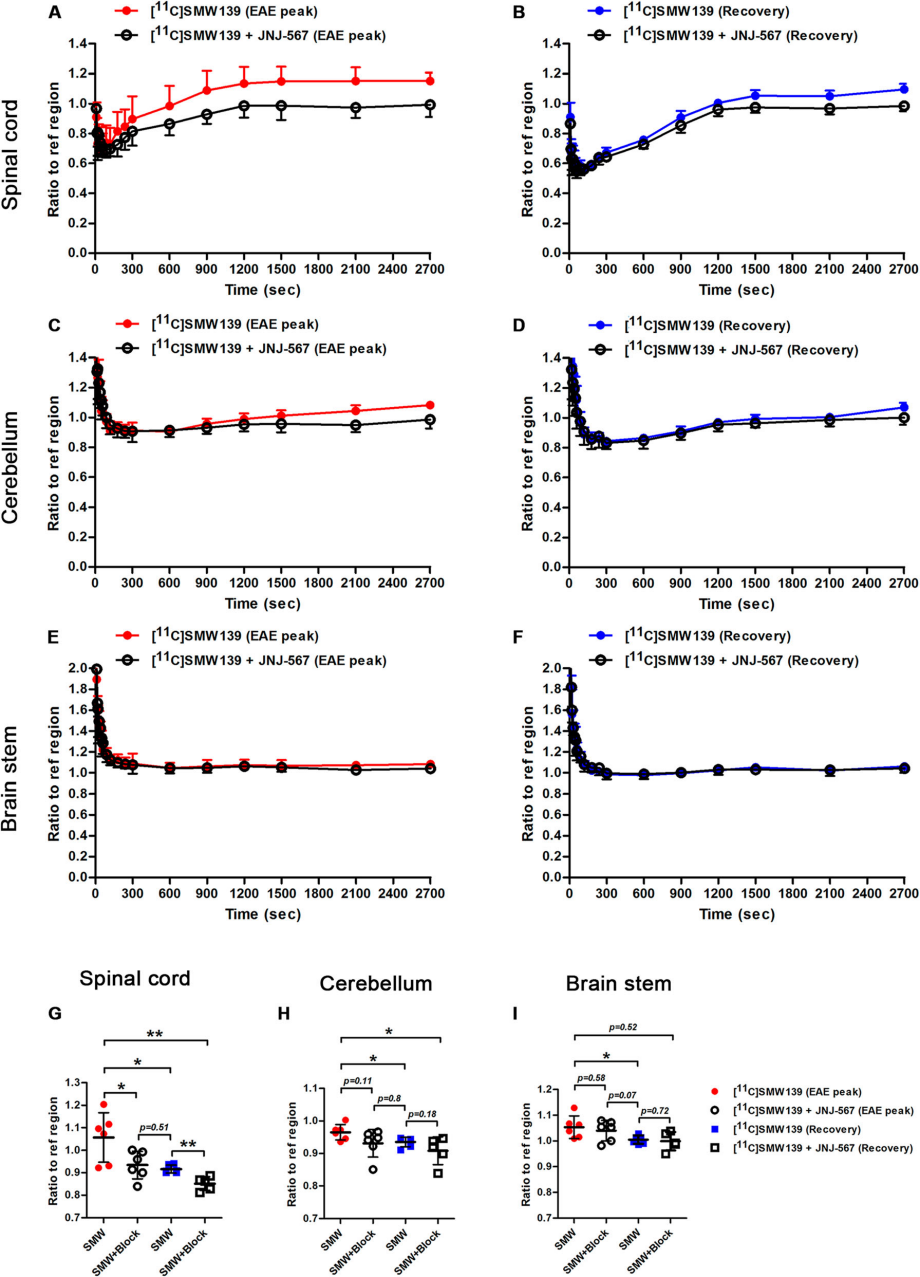
Specific blocking of [^11^C]SMW139 uptake in the brain of EAE animals at the peak of the disease and in the recovery phase. Time activity curves showing the ratio to reference region of [^11^C]SMW139 uptake in the spinal cord (**a**, **b**), cerebellum (**c**, **d**), and brain stem (**e**, **f**) before and after blocking with JNJ-47965567 (30 mg/kg) at the peak of the disease and in the recovery phase. Ratio to reference region of [^11^C]SMW139 uptake in spinal cord (**g**), cerebellum (**h**), and brain stem (**i**) of a single-frame static reconstruction between 5 and 45 min at the peak of the disease and in the recovery phase. (*, *p* < 0.05; **, *p* < 0.001)

We observed a significant blocking of the tracer uptake in the spinal cord of the animal at the peak of the disease (Fig. 6a). A less pronounced blocking effect was detected in the cerebellum and brain stem (Fig. 6c–e). In the recovery phase, we still observed a slight blocking effect in the spinal cord but not in the cerebellum and brain stem (Fig. 6b, d, f) (Additional file 1, Fig. 8).

Quantification of static scans showed significantly lower spinal cord to reference region ratio for the blocking scan compared to the non-block at the peak of the disease (0.93 ± 0.06 vs 1.06 ± 0.1, *p* < 0.05; respectively) and in the recovery phase (0.85 ± 0.03 vs 0.91 ± 0.02, *p* < 0.001; respectively) (Fig. 6g). We also observed lower but not significant cerebellum to reference region ratio for the blocking scan compared to the non-block at the peak of the disease (0.93 ± 0.04 vs 0.97 ± 0.02, *p* = 0.11; respectively) and in the recovery phase (0.91 ± 0.04 vs 0.93 ± 0.01, *p* = 0.18; respectively) (Fig. 6h). We did not observe a significant blocking effect in the brain stem of the animal at the peak of the disease or in the recovery phase (Fig. 6i).

### Correlation of [^11^C]SMW139 uptake with EAE clinical scores

To determine if PET imaging with [^11^C]SMW139 tracer could give an indication on the severity of the neuroinflammation, we investigated a possible correlation between the uptake of the [^11^C]SMW139 tracer and the severity of the EAE clinical outcome in rats. In our EAE cohorts, we had animals that developed different level of clinical symptoms and were imaged with [^11^C]SMW139 at the peak of the disease and when they fully recovered. The difference in [^11^C]SMW139 uptake between the scan at the peak of the disease and the recovery phase should represent the level of neuroinflammation and was correlated with the clinical score of the animal at the scanning time point.

**Fig. 7:**
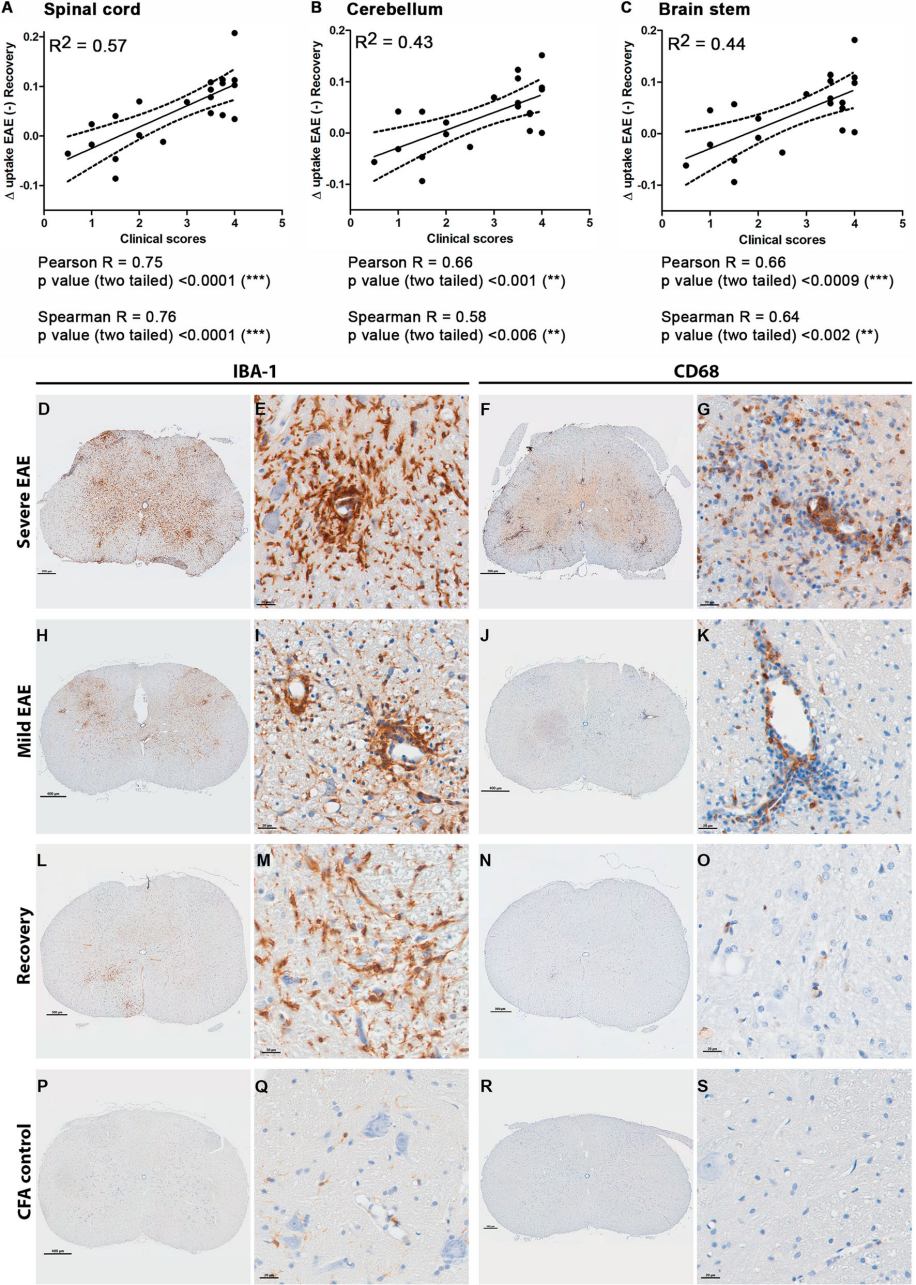
Correlation of the EAE clinical score with the uptake of [^11^C]SMW139 in the brain and spinal cord and immunohistochemistry staining on spinal cord tissue from EAE animals and controls. Graphs showing the correlation of the difference of [^11^C]SMW139 uptake between the peak of the EAE disease and the recovery phase with the clinical scores of the same respective animals in the spinal cord (**a**), cerebellum (**b**), and brain stem (**c**). The goodness of the correlation was evaluated using Pearson and Spearman correlation factors and *p* value for significance. (**, *p* < 0.001; ***, *p* < 0.0001). Images showing IBA-1 and CD68 staining on spinal cord tissue from severe EAE rats (**d**–**g)**, mild EAE rats (**h**–**k**), recovery rats (**l**–**o**), and CFA control rats (**p**–**s**)

We observed a positive correlation in the spinal cord between the uptake of the [^11^C]SMW139 tracer and the clinical scores of the animals (*R*^2^ = 0.57; Pearson *R* = 0.75, *p* < 0.0001 Spearman *R* = 0.76 *p* < 0.0001) (Fig. 7a). In the cerebellum and brain stem, we also observed a positive correlation, but less strong, compared to the spinal cord (Fig. 7b, c).

### Autoradiography and immunohistochemistry

To validate the specificity of [^11^C]SMW139 tracer binding to the EAE tissue and validating the correlation between tracer uptake and severity of neuroinflammation, we performed autoradiography and immunohistochemistry on brain and spinal cord tissues of EAE animals.

In the severe EAE tissue, we observed a high expression of IBA-1 showing high activation level of microglia and massive infiltration of macrophages stained with CD68. The IBA-1 and CD68 staining was less pronounced in the tissues from mild EAE animals. In tissues from some animals that fully recovered, we still observed foci with high expression of IBA-1, indicating that activated microglia are still present. IBA-1 staining on tissues from CFA immunized rats showed low staining on ramified microglia with no pattern of activation, and no CD68-positive cells were observed (Fig 7d–s) (Additional file 1, Figures 5 and 6).

**Fig. 8:**
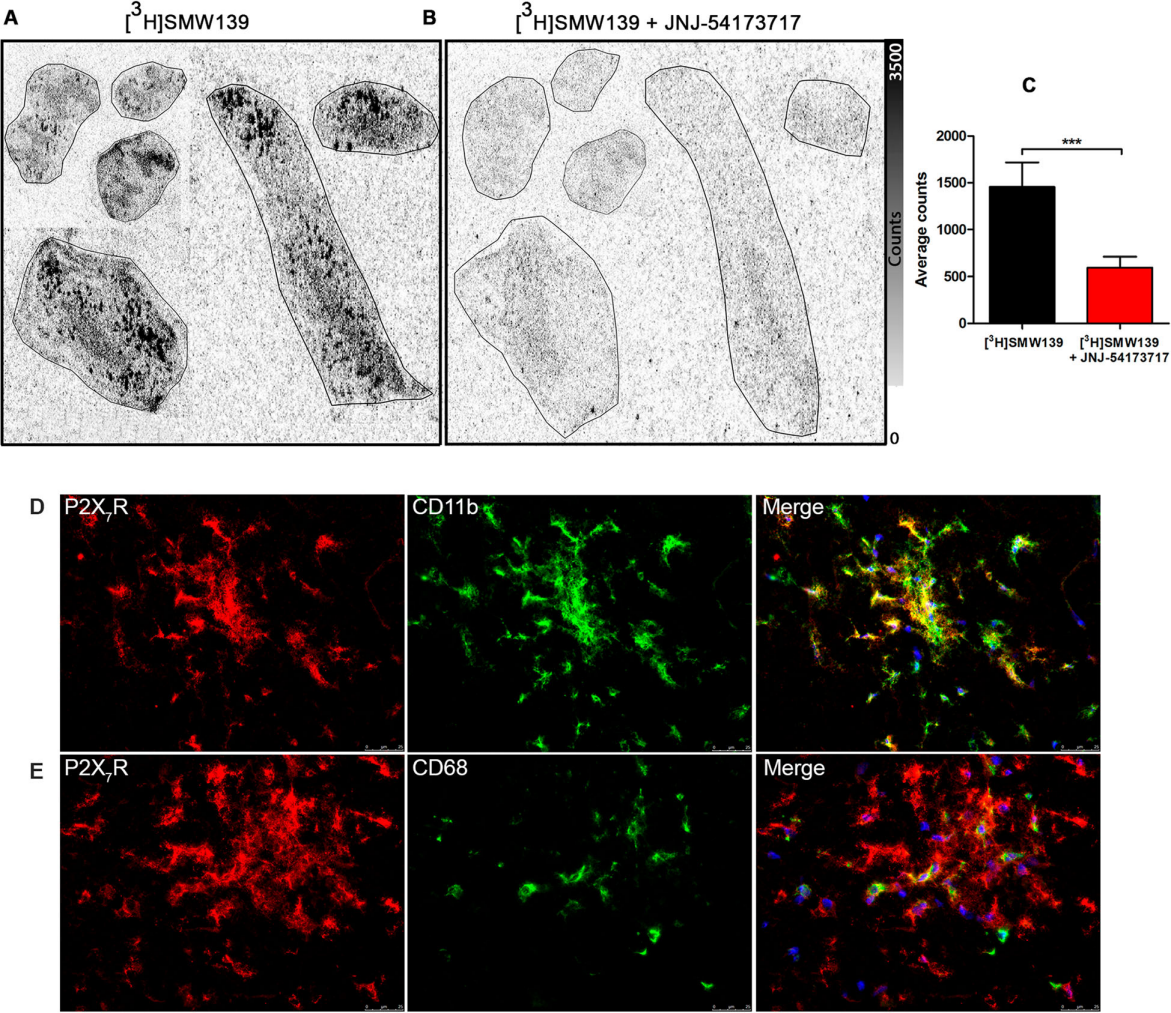
Autoradiography with [^3^H]SMW139 and immunofluorescence staining on spinal cord tissue from EAE animals at the peak of the disease. Autoradiograph showing the binding of [^3^H]SMW139 on spinal cord tissue from EAE animals at the peak of the disease (**a**) and blocking with JNJ-54173717 (**b**). Graph showing the autoradiography quantification of the [^3^H]SMW139 total binding and block (*n* = 3 different animals, *n* = 26 different sections) (**c**). Images are showing P2X_7_R (red) and CD11b (green) double staining (**d**) and P2X_7_R (red) and CD68 (green) double staining (**e**)

For autoradiography, we observed a significant binding of [^3^H]SMW139 to the EAE spinal cord tissue that was specifically blocked with co-incubation with JNJ-54173717 (Fig. 8a–c). The binding pattern of [^3^H]SMW139 showed focal high binding spots that correlated with neuroinflammation and infiltration foci in the spinal cord. Double staining for P2X_7_R and microglia markers (CD11b and CD68) showed colocalization of the staining and confirmed the expression of the target on the activated microglia (Fig. 8d, e).

To further validate the specificity of the [^11^C]SMW139 uptake in the brain, we performed ex vivo immunostaining on brain and spinal cord tissues collected immediately post-PET imaging acquisition in EAE animals at the peak of the disease (Additional file 1, Figure 10). We correlated the uptake of [^11^C]SMW139 in the PET images with the IBA-1, CD11b, CD68 and P2X_7_R staining in the respective brain and spinal cord areas. We observed a very good correlation between the uptake of the [^11^C]SMW139 in the brain and spinal cord of the EAE animal visualized by the PET images with the activated microglia stained with IBA-1, CD11b and CD68 at the same brain location. We also observe high P2X_7_R expression on activated microglia that correlates with the uptake of [^11^C]SMW139 in the PET images (Additional file 1, Figure 11–14).

## Discussion

The need for novel PET tracers for imaging microglia activation is not limited to multiple sclerosis but is also critical for other neurodegenerative disease like Alzheimer’s and Parkinson disease where recent studies demonstrate neuroinflammation involvement in their pathogeneses [25, 26]. Until now, TSPO ligands have been widely used in preclinical and clinical settings to evaluate neuroinflammation in vivo; however, their translation into routine clinical use has been hindered due to polymorphism and complicated quantification of specific binding of tracers [27, 28] in addition to their inability to differentiate between pro- and anti-inflammatory microglia. We previously showed that P2X_7_R is highly expressed in human pro-inflammatory microglia in MS lesions and in vitro [16]. Our data suggest that [^11^C]SMW139 is a potential PET tracer for imaging pro-inflammatory microglia giving new perspectives for better understanding of the relation between neuroinflammation and neurodegeneration.

P2X_7_R PET tracers are still in early phase of development, and some of them suffer from low BBB permeability [29, 30] or low affinity to rodent P2X_7_R [31] which can limit their utility and slow their translation to human. [^11^C]SMW139 shows good PET tracer characteristics with high affinity for P2X_7_R, good in vivo stability and ability to cross the BBB. The affinity for the rat P2X_7_R was fivefolds lower compared to the human P2X_7_R but remained in the acceptable nanomolar range for a PET tracer. Wilkinson et al. reported similar observation with lower binding affinity for comparable class of compounds (K_i_ and IC_50_ value) for mice compared to human P2X_7_R [24]. We have previously shown that [^11^C]SMW139 was able to enter the brain [21]; here we further validate that the tracer can freely diffuse across the BBB and that efflux pumps do not contribute to the clearance of the tracer. The in vivo stability of the tracer was also in accordance with published data by Janssen et al. [21] in naïve rats and here we show that stability and metabolism of the tracer is not affected by neuroinflammation processes.

To our knowledge, this is the first successful study to evaluate P2X_7_R PET tracers in the EAE model. Han et al. attempted to evaluate [^11^C]GSK1482160 in vivo in the EAE model but did not succeed due to the low affinity of the tracer for rat P2X_7_R [31]. Systemic and intracerebral LPS models have been used to evaluate the currently available P2X_7_R tracers [17, 30, 32]; however, these artificial models are not ideal and P2X_7_R overexpression may not reflect the real pathological expression levels in neuroinflammation. We used the acute EAE model in Lewis rats which is characterized by microglia activation and macrophage infiltration mainly in the spinal cord and to a lesser extent in the brain stem and cerebellum [33, 34]. This EAE model is more suitable for initial evaluation and validation of PET tracers for neuroinflammation compared to more complex demyelinating EAE models that can complicate the interpretation of the imaging results. In our hands, the EAE developed in Lewis rat was heterogeneous with animals showing low and high clinical scores. Only the severe-relapsing and severe acute animals showed higher uptake in the inflammatory phase compared to the recovery phase but not the mild acute animals. This is probably due to the low level of inflammation during the mild EAE.

A high inflammatory environment is predominant in the EAE peak phase where microglia activation status is tipped toward pro-inflammatory phenotype [34]. The uptake of [^11^C]SMW139 was higher in the spinal cord at the peak of the disease compared to the recovery phase. The higher tracer uptake is mainly driven by the overexpression of P2X_7_R on pro-inflammatory microglia and macrophages in the brain. Nonetheless, we cannot exclude that the increased density of these cells due to gliosis and infiltration of peripheral monocytes also contributed to the increased PET signal in the inflammatory brain parts. Immunohistochemical staining for IBA1 and CD68 support this hypothesis and shows that both highly activated microglia and infiltrated macrophage are present in the inflammatory tissue. In addition, the pattern of IBA1 and CD68 staining shows good correlation with the autoradiography results with [^3^H]SMW139.

Relapses are not common in the acute EAE in Lewis rats but occasionally occur following the initial EAE phase in certain animals [35]. In our experimental groups, relapses occurred in four animals of which two were imaged during the relapse phase. The increase in [^11^C]SMW139 uptake in the cerebellum and brain stem in the relapse phase suggests an increase of neuroinflammation in these areas that can be responsible for the clinical relapse. The small number of animals does not allow drawing strong conclusions but gives an indication that [^11^C]SMW139 is capable of depicting neuroinflammation changes in the brain. The dynamics of neuroinflammation during the relapse phase need to be further evaluated in a more robust relapsing-remitting or chronic EAE model.

For visualization of microglial activation, tracers binding to the TSPO are mostly used. A significant number of clinical studies have been devoted to the evaluation of TSPO tracers in MS [28] and showed higher uptake in normal appearing white matter and T2 lesions of MS patients compared to control healthy volunteers [36–38], but limited studies showed correlation with disability (EDSS) [37, 39, 40]. Despite the established role for TSPO-PET imaging in detecting activated microglia in vivo, it had minimal impact on the understanding of the disease progression and treatment monitoring. In addition, TSPO tracers suffer from several challenges like low signal-to-noise ratio, sensitivity to genetic polymorphism, expression of TSPO on endothelial cells at the BBB and astrocytes, and the inability to differentiate between pro- and anti-inflammatory phenotype of activated microglia. To that end, targeting P2X_7_R could provide a new perspective in imaging neuroinflammation, and our current evaluation showed that [^11^C]SMW139 is a good candidate PET tracer for imaging neuroinflammation. The uptake of the tracer correlated well with the clinical scores of the EAE animal and with the level of neuroinflammation, based on the in vivo PET imaging and immunostaining results. Recently, Hagens et al. reported a first in man PET study with [^11^C]SMW139 and showed that the uptake or the tracer can be quantified with PET using BP_ND_ as a measure for specific binding in healthy controls and relapsing-remitting MS patients [41].

## Conclusion

In conclusion, our data show that [^11^C]SMW139 is a promising PET tracer for imaging neuroinflammation and evaluating the dynamic changes of pro-inflammatory microglia in the brain. This may help in obtaining insights into the role of microglia in disease progression and enabling the development of novel treatment strategies aimed at modulating the immune response in order to promote neuroprotection.

## Supplementary information

**Additional file 1: Supp. Figure 1.** Figure showing the region of interest drawn for the PET scans analysis. Vivoquant atlas was applied to delineate the brain stem and cerebellum, and manual region of interest was drawn on the spinal cord (Shown in red in the lower panel). **Supp. Figure 2.** Time activity curve of [11C]SMW139 uptake in the spinal cord (%ID/mL) at the peak of the EAE disease comparing the different EAE severity groups and CFA control on the same graph. **Supp. Figure 3.** Time activity curves of [11C]SMW139 uptake (%ID/mL) in the spinal cord (A), cerebellum (B), brain stem (C) of the rat immunized for EAE but did not develop clinical symptoms. PET imaging was performed at day 14 (EAE peaks) (●) and day 35 (Recovery) (■) post-immunization. **Supp. Figure 4.** Time activity curve of [11C]SMW139 uptake in the reference region (forebrain) at the peak of the EAE (●) and after full recovery from the EAE clinical signs (■) showing no difference in [11C]SMW139 uptake in the reference region between the two phases of the disease. **Supp. Figure 5.** Immunohistochemistry staining of IBA-1 and CD68 (ED1) on spinal cord longitudinal sections of severe EAE rats showing inflammation and infiltration of macrophages/monocytes in several locations in the cerebellum and brain stem. The staining shows focal foci of neuroinflammation which mirror the pattern of [3H]SMW139 binding to the EAE spinal cord tissue in autoradiography. **Supp. Figure 6.** Immunohistochemistry staining of IBA-1 and CD68 (ED1) in the cerebellum of severe EAE rats showing inflammation and infiltration of macrophages/monocytes in several locations in the cerebellum and brain stem. **Supp. Figure 7.** PET-Gadolinium MRI T1 scan in severe EAE rats at the peak of the disease showing T1 enhancement signal in the spinal cord which co-localize with an increase of [11C]SMW139 uptake in the same area (A-D). When the animal are fully recovered no gadolinium enhancement is observed in the brain and the uptake of [11C]SMW139 is significantly lower. **Supp. Figure 8.** Time activity curves of the ratio to reference region of [11C]SMW139 uptake in spinal cord at the peak of the EAE (●) and after full recovery from EAE clinical symptoms (■). **Supp. Figure 9.** PET images of [11C]SMW139 in the EAE rats at the peak of the disease and in the recovery phase. Sagittal, coronal and axial cross sections PET images showing [11C]SMW139 uptake in the brain and spinal cord of severe-relapsing, severe acute, mild acute EAE, and CFA control. **Supp. Figure 10.** Experimental plan of PET imaging with [11C]SMW139 and ex vivo validation (A); Clinical scores and weight of the EAE animals (n=4) (B); Representative PET images of [11C]SMW139 in the EAE rats at the peak of the disease. The green and red spots in the brain and spinal cord indicate a high accumulation of [11C]SMW139 (C). **Supp. Figure 11.** Correlation between uptake of [11C]SMW139 tracer in the brain of the EAE animal and the ex vivo immunostaining for IBA-1 and ED-1; Transversal (A) and sagittal (D) PET image section showing the uptake of the [11C]SMW139 in the brain. The dotted purple circles or rectangles mark the area with the highest uptake. The green and red spots in the brain indicate a high accumulation of [11C]SMW139; Immunostaining with IBA-1 (B, E) and CD68 (C, F) of the respective brain region post PET imaging showing high microglia activation in the same region where the high uptake of [11C]SMW139 was observed by PET imaging. **Supp. Figure 12.** Correlation between uptake of [11C]SMW139 tracer in the spinal cord of the EAE animal and the ex vivo immunostaining for IBA-1 and ED-1; Sagittal (A) and transversal (C) PET image section showing the uptake of the [11C]SMW139 in the spinal cord. The dotted red circles or rectangles mark the area with the highest uptake. The green and red spots in the spinal cord indicate a high accumulation of [11C]SMW139 (A,C); Immunostaining with IBA-1 and CD68 (B, D) of the respective brain region post PET imaging showing high microglia activation in the same region where the high uptake of [11C]SMW139 was observed by PET imaging. **Supp. Figure 13.** Correlation between uptake of [11C]SMW139 tracer in the spinal cord of the EAE animals and the ex vivo immunostaining for CD11b (microglia) and P2X7R; Sagittal (A) and transversal (C) PET image section showing the uptake of the [11C]SMW139 in the spinal cord. The dotted red circles or rectangles mark the area with the highest uptake. The green and red spots in the spinal cord indicate a high accumulation of [11C]SMW139 (A, C); Immunostaining with CD11b and P2X7R (B, D) of the respective brain region post PET imaging showing high microglia activation that expresses P2X7R in the same region where the high uptake of [11C]SMW139 was observed by PET imaging. **Supp. Figure 14.** Correlation between uptake of [11C]SMW139 tracer in the brain of the EAE animals and the ex vivo immunostaining for CD11b and P2X7R; Sagittal PET image section showing the uptake of the [11C]SMW139 in the brain. The area with the high uptake is marked by the doted red rectangle or circle. The green and red spots in the spinal cord indicate a high accumulation of [11C]SMW139 (A); Immunostaining with CD11b (microglia) and P2X7R (B, C) of the respective brain region post PET imaging showing high microglia activation in the same region where the high uptake of [11C]SMW139 was observed by PET imaging.

## Data Availability

All data generated or analyzed during this study are included in this published article and its supplementary information files.

## Abbreviations

FBS: Fetal bovine serum
RPM: Round per minute
RT: Room temperature
EAE: Experimental autoimmune encephalomyelitis
CFA: Complete Freund’s adjuvant
RPM: Round per minute
ROI: Region of interest
MS: Multiple sclerosis
PET: Positron emission tomography
MR: Magnetic resonance
CT: Computed tomography
MBP: Myelin basic protein
TSPO: Translocator protein
TAC: Time activity curve
AUC: Area under the curve
