# Supplementary material for: PET imaging of P2X7R in the experimental autoimmune encephalomyelitis model of multiple sclerosis using [11C]SMW139
Source: J Neuroinflammation. 2020 Oct 14;17:300. doi: 10.1186/s12974-020-01962-7 (PMC7556947; doi:10.1186/s12974-020-01962-7)
Supplement: Supplementary file 1 — Additional file 1: Supp. Figure 1. Figure showing the region of interest drawn for the PET scans analysis. Vivoquant atlas was applied to delineate the brain stem and cerebellum, and manual region of interest was drawn on the spinal cord (Shown in red in the lower panel). Supp. Figure 2. Time activity curve of [11C]SMW139 uptake in the spinal cord (%ID/mL) at the peak of the EAE disease comparing the different EAE severity groups and CFA control on the same graph. Supp. Figure 3. Time activity curves of [11C]SMW139 uptake (%ID/mL) in the spinal cord (A), cerebellum (B), brain stem (C) of the rat immunized for EAE but did not develop clinical symptoms. PET imaging was performed at day 14 (EAE peaks) (●) and day 35 (Recovery) (■) post-immunization. Supp. Figure 4. Time activity curve of [11C]SMW139 uptake in the reference region (forebrain) at the peak of the EAE (●) and after full recovery from the EAE clinical signs (■) showing no difference in [11C]SMW139 uptake in the reference region between the two phases of the disease. Supp. Figure 5. Immunohistochemistry staining of IBA-1 and CD68 (ED1) on spinal cord longitudinal sections of severe EAE rats showing inflammation and infiltration of macrophages/monocytes in several locations in the cerebellum and brain stem. The staining shows focal foci of neuroinflammation which mirror the pattern of [3H]SMW139 binding to the EAE spinal cord tissue in autoradiography. Supp. Figure 6. Immunohistochemistry staining of IBA-1 and CD68 (ED1) in the cerebellum of severe EAE rats showing inflammation and infiltration of macrophages/monocytes in several locations in the cerebellum and brain stem. Supp. Figure 7. PET-Gadolinium MRI T1 scan in severe EAE rats at the peak of the disease showing T1 enhancement signal in the spinal cord which co-localize with an increase of [11C]SMW139 uptake in the same area (A-D). When the animal are fully recovered no gadolinium enhancement is observed in the brain and the uptake of [11C]SMW139 is sig [file 12974_2020_1962_MOESM1_ESM.docx]

**Additional file 1**

**Supp. Figure 1.**

Figure showing the region of interest drawn for the PET scans analysis. Vivoquant atlas was applied to delineate the brain stem and cerebellum, and manual region of interest was drawn on the spinal cord (Shown in red in the lower panel)


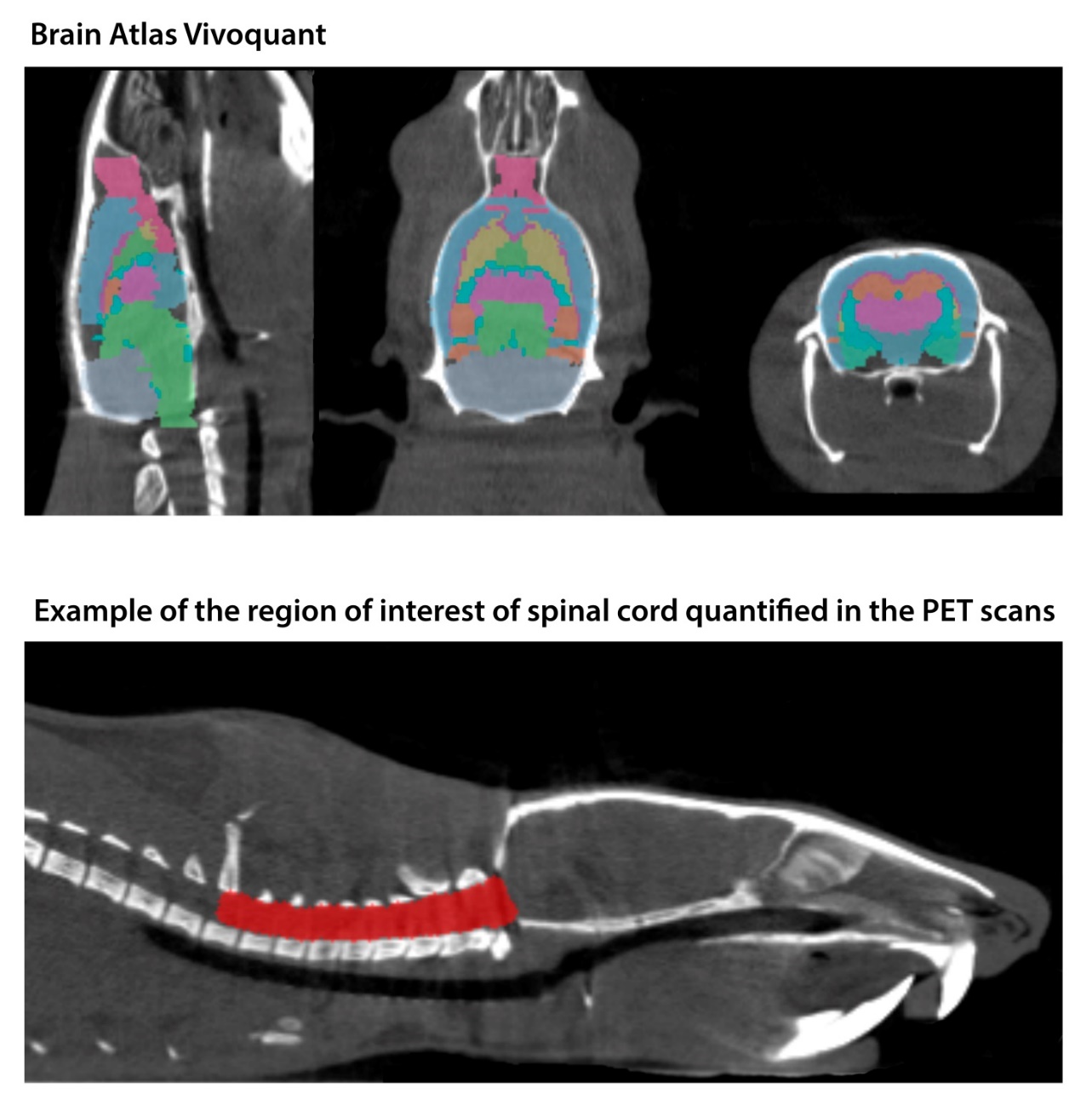


**Legend:** Region of interest drawn for the PET scans analysis.

**Supp. Figure 2.**


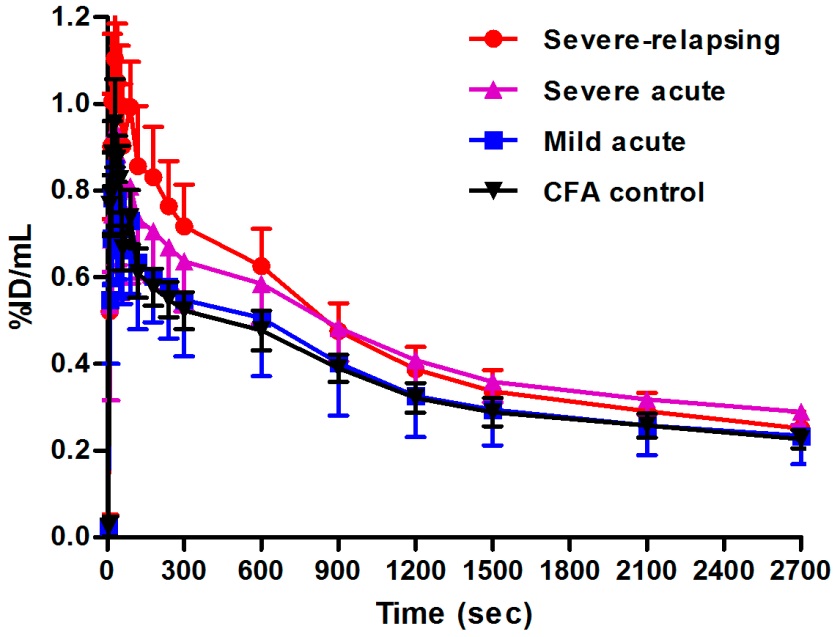


**Legend:** Time activity curve of [^11^C]SMW139 uptake in the spinal cord at the peak of the EAE disease comparing the different EAE severity groups and CFA control on the same graph.

**Supp. Figure 3.**

Time activity curves of [^11^C]SMW139 uptake (%ID/mL) in the spinal cord (A), cerebellum (B), brain stem (C) of the rat immunized for EAE but did not develop clinical symptoms. PET imaging was performed at day 14 (EAE peaks) (●) and day 35 (Recovery) (■) post-immunization.

**A**

**
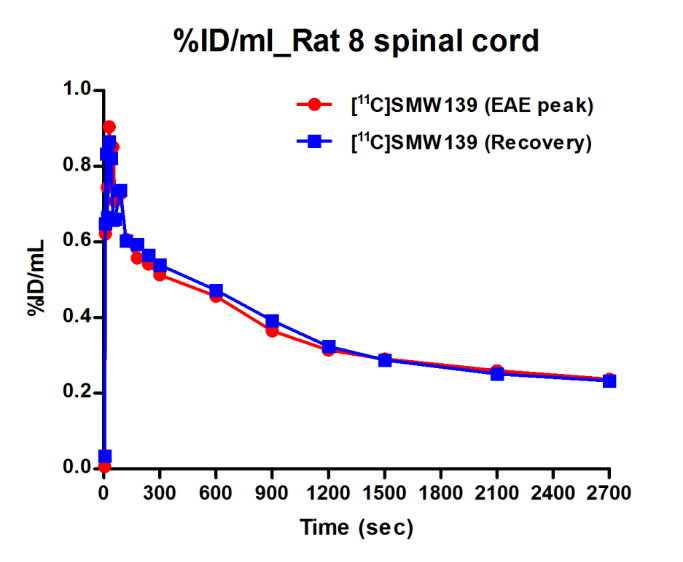

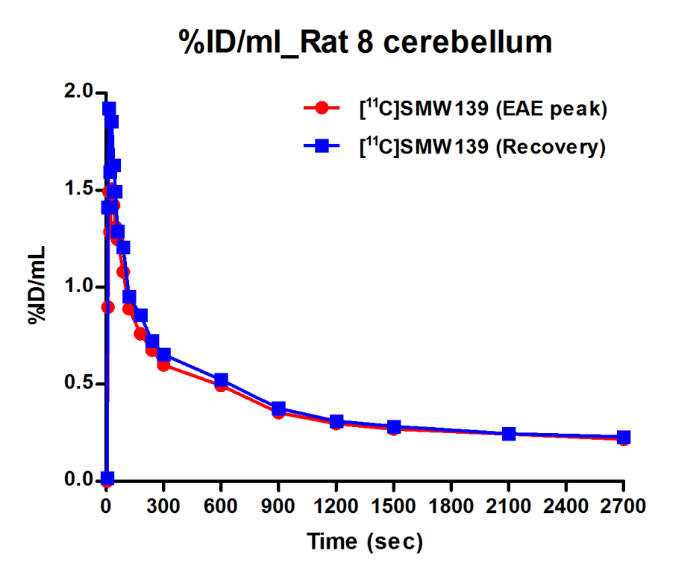
**

**B**

**C**

**
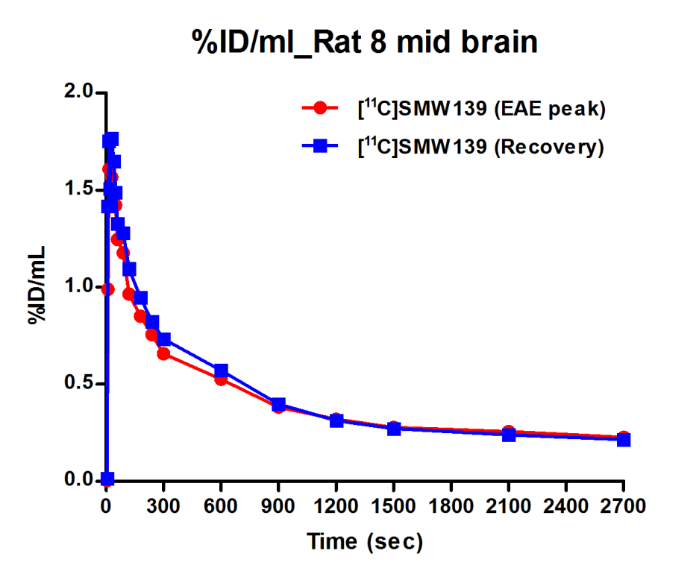
**

**Legend:** Time activity curves of [^11^C]SMW139 uptake in the rat immunized for EAE but did not develop clinical symptoms.

**Supp. Figure 4.**

Time activity curve of [^11^C]SMW139 uptake in the reference region (forebrain) at the peak of the EAE (●) and after full recovery from the EAE clinical signs (■) showing no difference in [^11^C]SMW139 uptake in the reference region between the two phases of the disease.


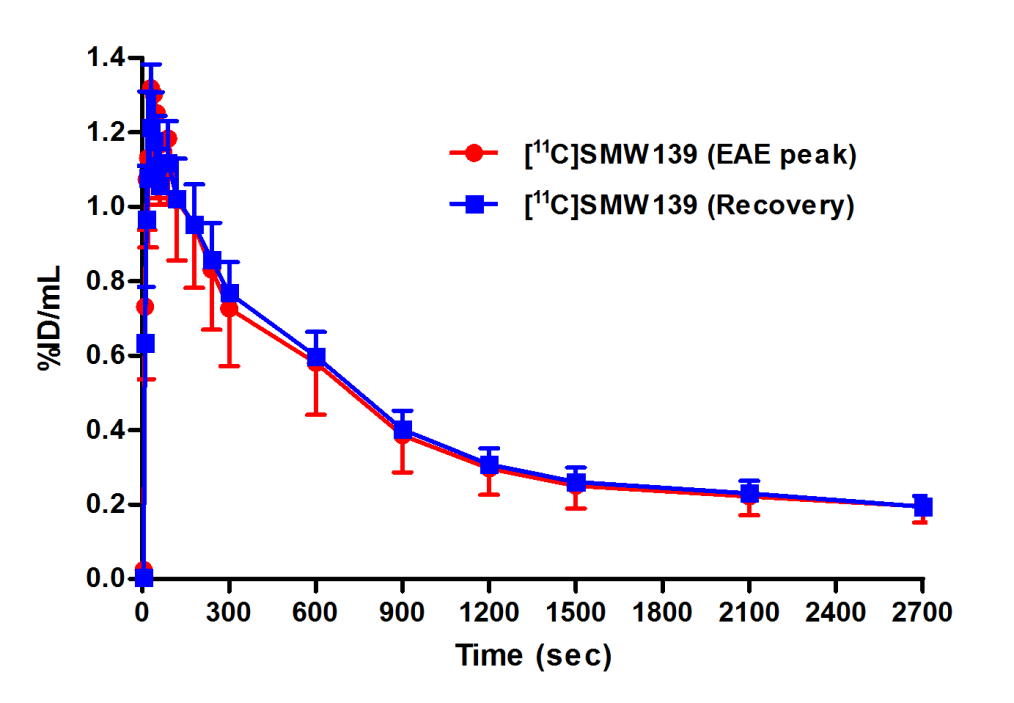


**Legend:** Time activity curve of [^11^C]SMW139 uptake in the reference region.

**Supp. Figure 5.**

Immunohistochemistry staining of IBA-1 and CD68 (ED1) on spinal cord longitudinal sections of severe EAE rats showing inflammation and infiltration of macrophages/monocytes in several locations in the cerebellum and brain stem. The staining shows focal foci of neuroinflammation which mirror the pattern of [^3^H]SMW139 binding to the EAE spinal cord tissue in autoradiography.


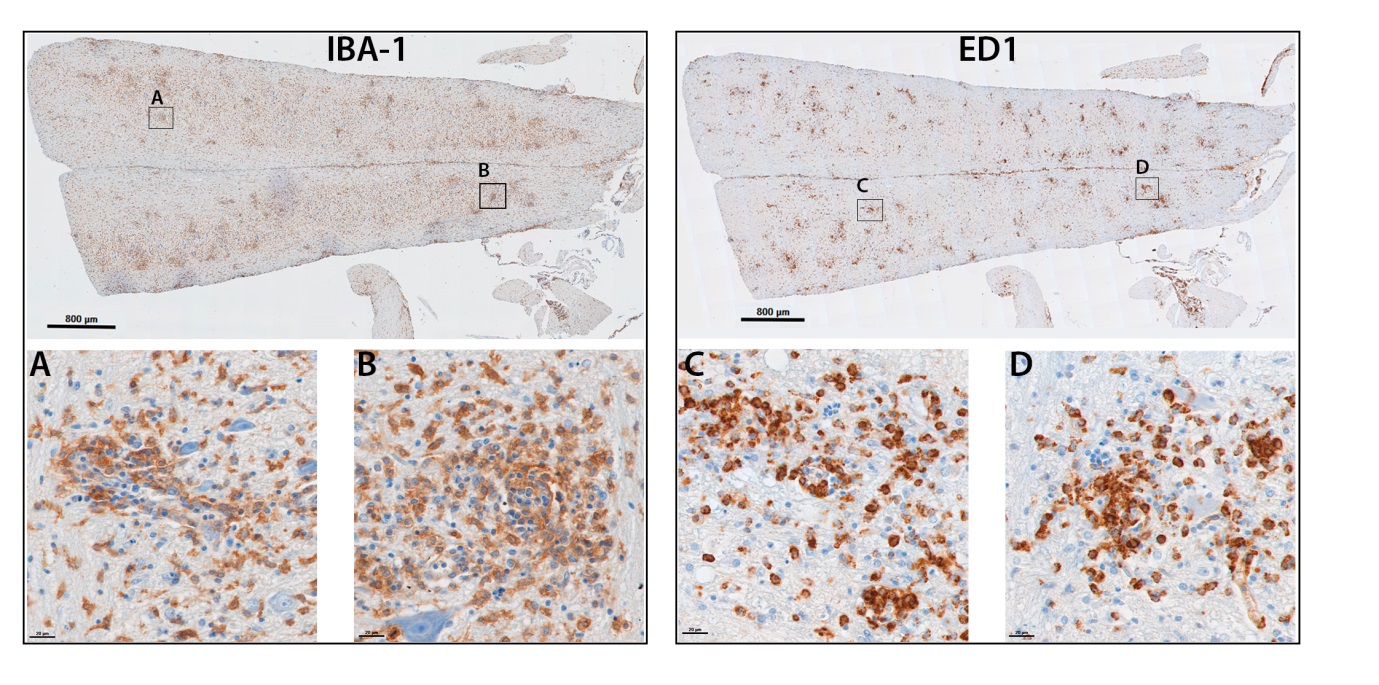


**Legend:** Immunohistochemistry staining of IBA-1 and CD68 (ED1) on spinal cord longitudinal sections of severe EAE rats.

**Supp. Figure 6.**

Immunohistochemistry staining of IBA-1 and CD68 (ED1) in the cerebellum of severe EAE rats showing inflammation and infiltration of macrophages/monocytes in several locations in the cerebellum and brain stem.


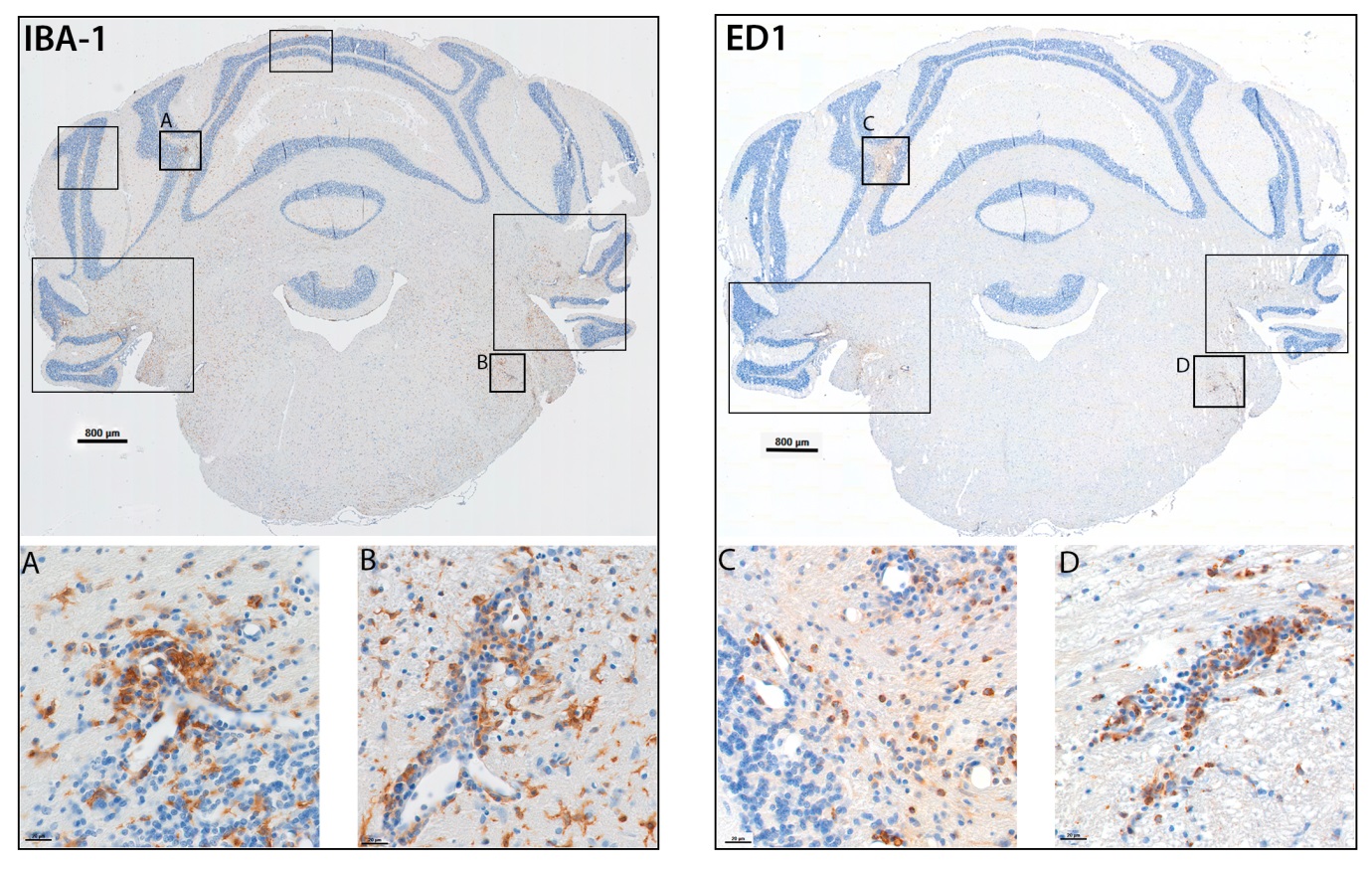


**Legend:** Immunohistochemistry staining of IBA-1 and CD68 (ED1) in the cerebellum of severe EAE rats.

**Supp. Figure 7.**

PET-Gadolinium MRI T1 scan in severe EAE rats at the peak of the disease showing T1 enhancement signal in the spinal cord which co-localize with an increase of [^11^C]SMW139 uptake in the same area (A-D). When the animal are fully recovered no gadolinium enhancement is observed in the brain and the uptake of [^11^C]SMW139 is significantly lower (E-H).


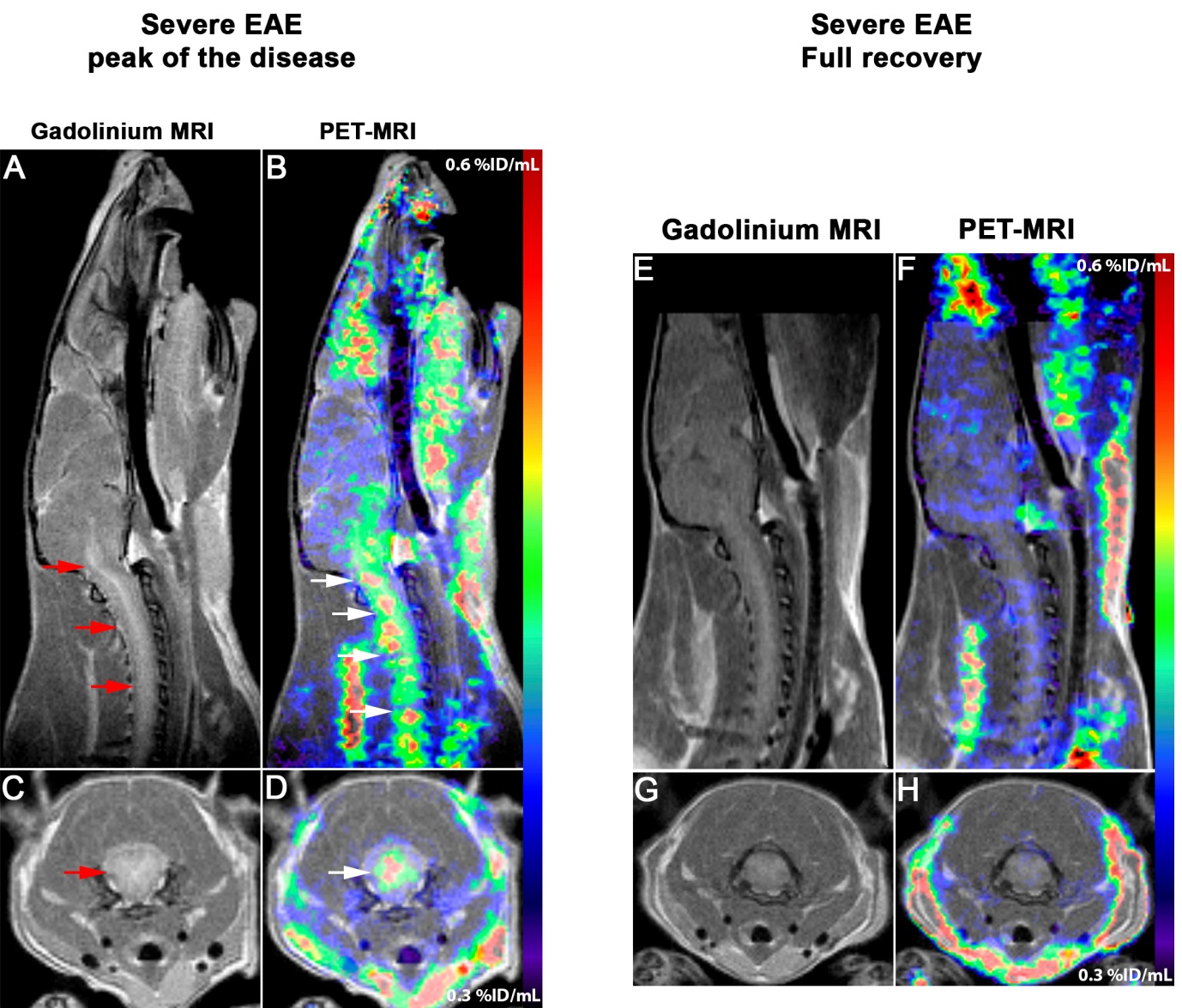


**Legend:** [^11^C]SMW139 PET and Gadolinium MRI T1 scan in severe EAE rats at the peak of the disease.

**Supp. Figure 8.**


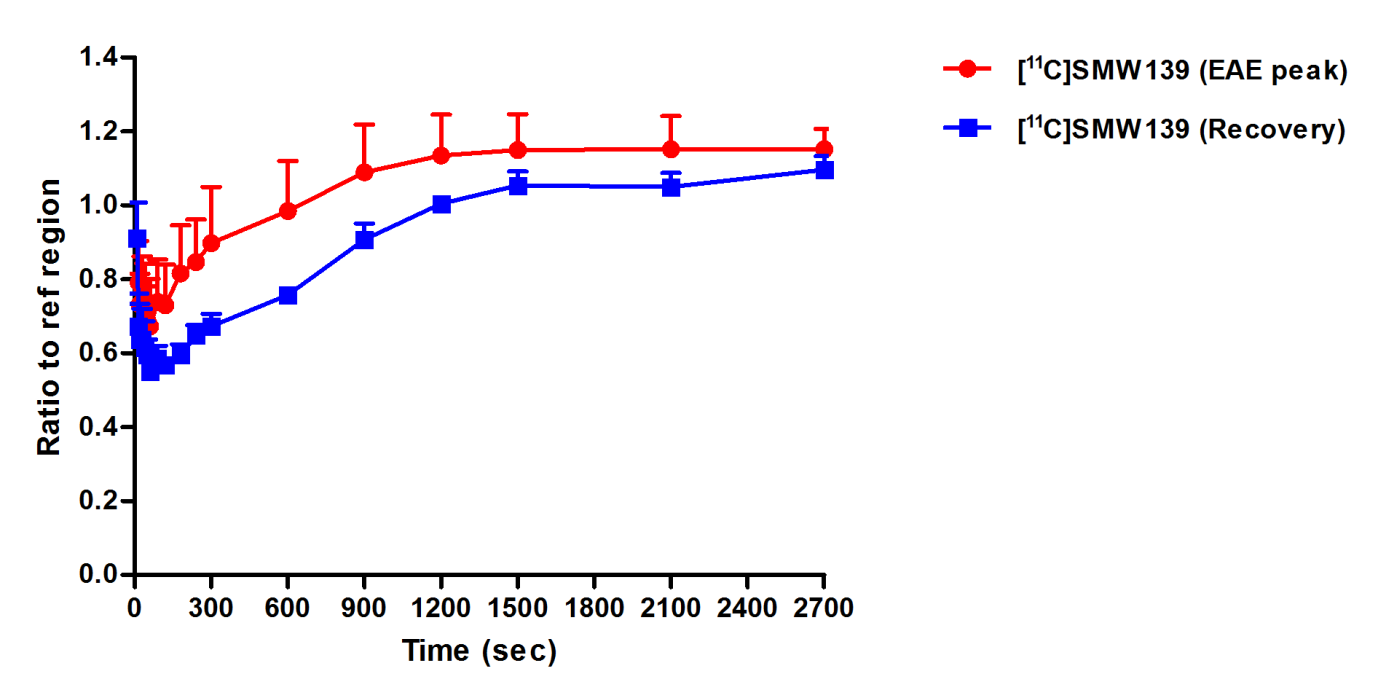


**Legend:** Time activity curves of the ratio to reference region of [^11^C]SMW139 uptake in spinal cord at the peak of the EAE and after full recovery from EAE clinical symptoms.

**Supp. Figure 9.**

PET images of [^11^C]SMW139 in the EAE rats at the peak of the disease and in the recovery phase. Sagittal, coronal and axial cross sections PET images showing [^11^C]SMW139 uptake in the brain and spinal cord of severe-relapsing, severe acute, mild acute EAE, and CFA control.


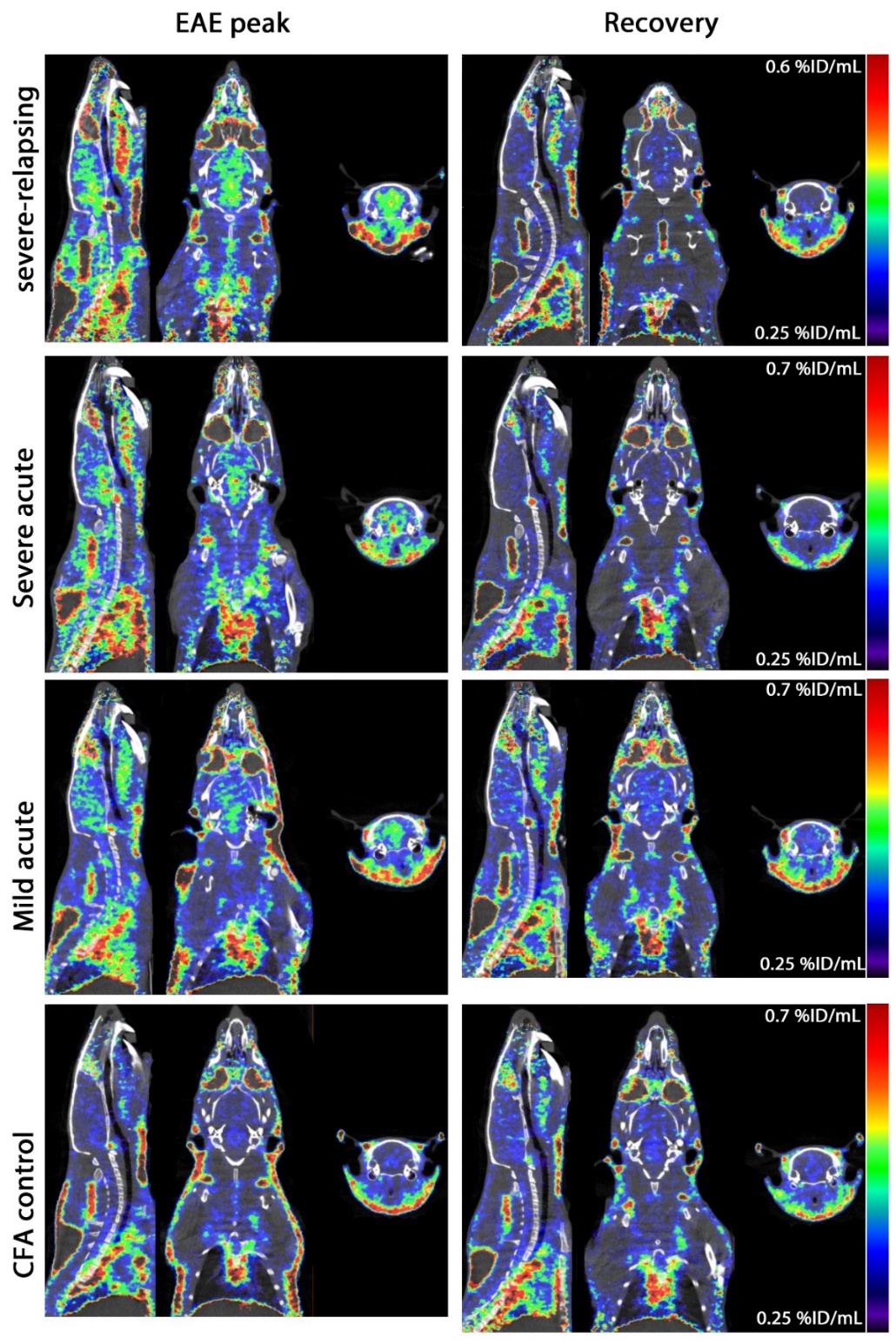


**Legend:** PET images of [^11^C]SMW139 in the EAE rats at the peak of the disease and in the recovery phase.

**Additional experiment upon reviewer’s request**

As the reviewer suggested we performed an additional experiment in the EAE animals where we imaged the rats at the peak of the disease with [^11^C]SMW139, followed by sacrificing the animals and immunostaining for Iba1, CD68 and P2X_7_R. We correlated the immunostaining with the PET images. The study design, results, and figures are shown below (Figure 10 to 14).

We induced EAE in female Lewis rats (n=4) as described in the manuscript. At the peak of the disease (day 12-13) we performed PET imaging with [^11^C]SMW139 (15 -25 MBq). Image acquisition and reconstruction was performed as described in the manuscript. Immediately after PET imaging acquisition, we sacrificed the animal, brain and spinal cord were collected and fixed with PFA 4% (n=2) or snap frozen (n=2). Tissue were then processed and cut in different orientation to allow correlation with the uptake of the tracer observed in PET images (Figure 10).

We performed immunostaining for IBA-1, CD68 on paraffin-embedded section and for P2X_7_R and CD11b on frozen sections using the same protocols described in the manuscript.

The results show a very good correlation between the uptake of the [^11^C]SMW139 in the brain and spinal cord of the EAE animal visualized in the PET images with the IBA-1, CD68 and P2X_7_R ex vivo staining at the same brain location **(supplementary Figure 11 to 14)**. These results validate the specificity of the [^11^C]SMW139 targeting and prove its capability of imaging neuroinflammation in the brain.

**Supp. Figure 10.**

Experimental plan of PET imaging with [^11^C]SMW139 and ex vivo validation (A); Clinical scores and weight of the EAE animals (n=4) (B); Representative PET images of [^11^C]SMW139 in the EAE rats at the peak of the disease. The green and red spots in the brain and spinal cord indicate a high accumulation of [^11^C]SMW139 (C)


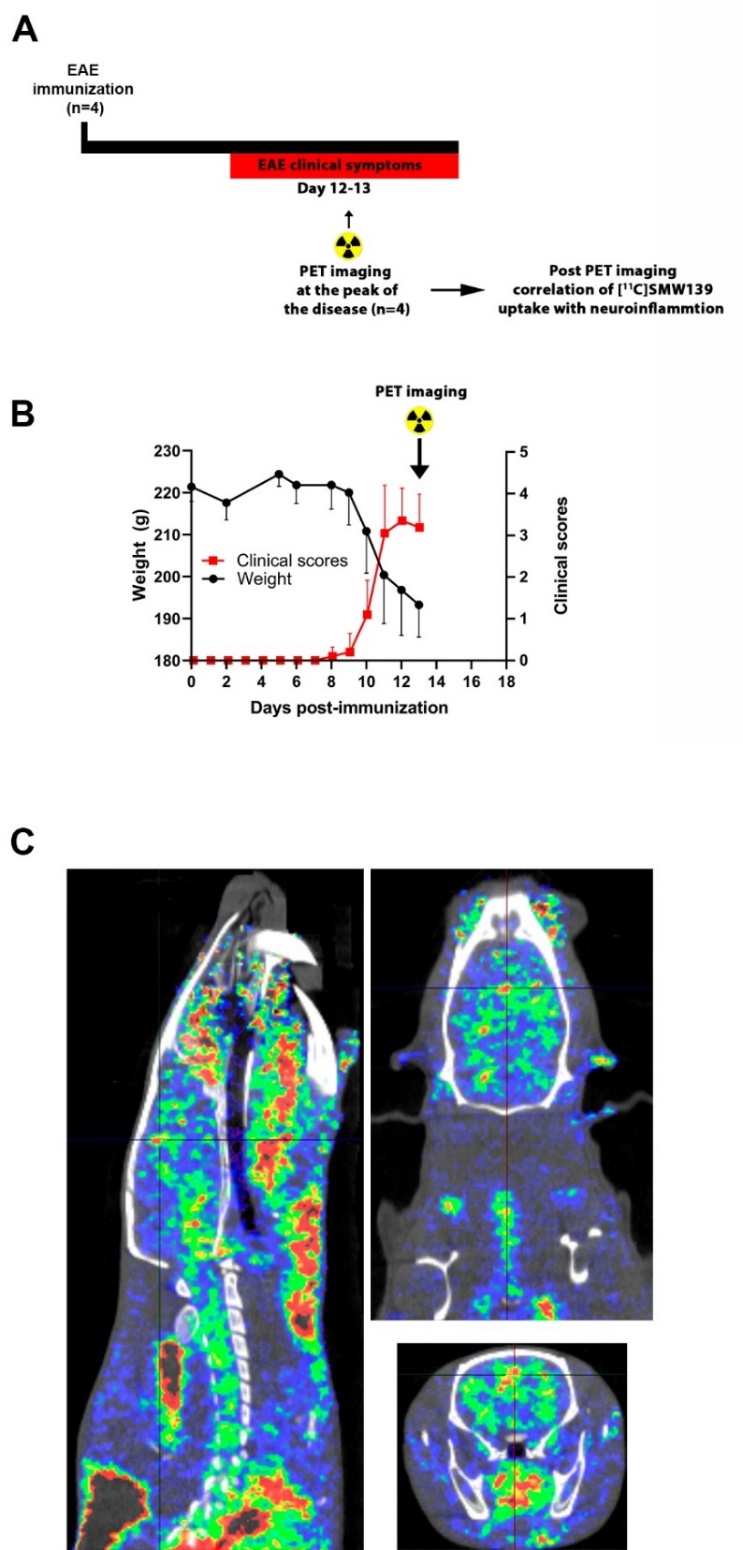


**Legend:** Experimental plan of PET imaging with [^11^C]SMW139 and ex vivo validation.

**Supp. Figure 11.**

Correlation between uptake of [^11^C]SMW139 tracer in the brain of the EAE animal and the ex vivo immunostaining for IBA-1 and ED-1; Transversal (A) and sagittal (D) PET image section showing the uptake of the [^11^C]SMW139 in the brain. The dotted purple circles or rectangles mark the area with the highest uptake. The green and red spots in the brain indicate a high accumulation of [^11^C]SMW139; Immunostaining with IBA-1 (B, E) and CD68 (C, F) of the respective brain region post PET imaging showing high microglia activation in the same region where the high uptake of [^11^C]SMW139 was observed by PET imaging.


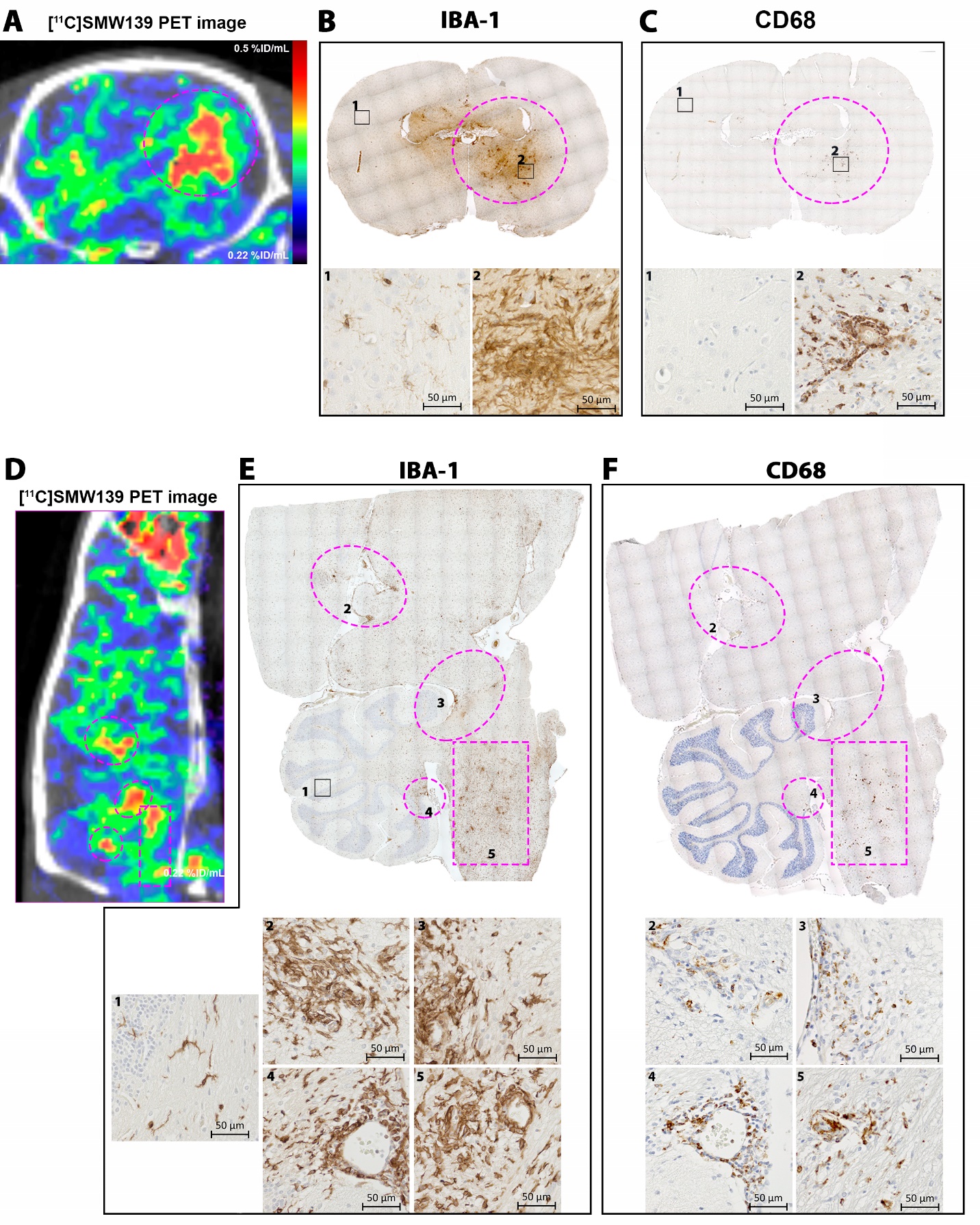


**Legend:** Correlation between uptake of [^11^C]SMW139 tracer in the brain of the EAE animals and the ex vivo immunostaining for IBA-1 and ED-1.

**Supp. Figure 12.**

Correlation between uptake of [^11^C]SMW139 tracer in the spinal cord of the EAE animal and the ex vivo immunostaining for IBA-1 and ED-1; Sagittal (A) and transversal (C) PET image section showing the uptake of the [^11^C]SMW139 in the spinal cord. The dotted red circles or rectangles mark the area with the highest uptake. The green and red spots in the spinal cord indicate a high accumulation of [^11^C]SMW139 (A,C); Immunostaining with IBA-1 and CD68 (B, D) of the respective brain region post PET imaging showing high microglia activation in the same region where the high uptake of [^11^C]SMW139 was observed by PET imaging.


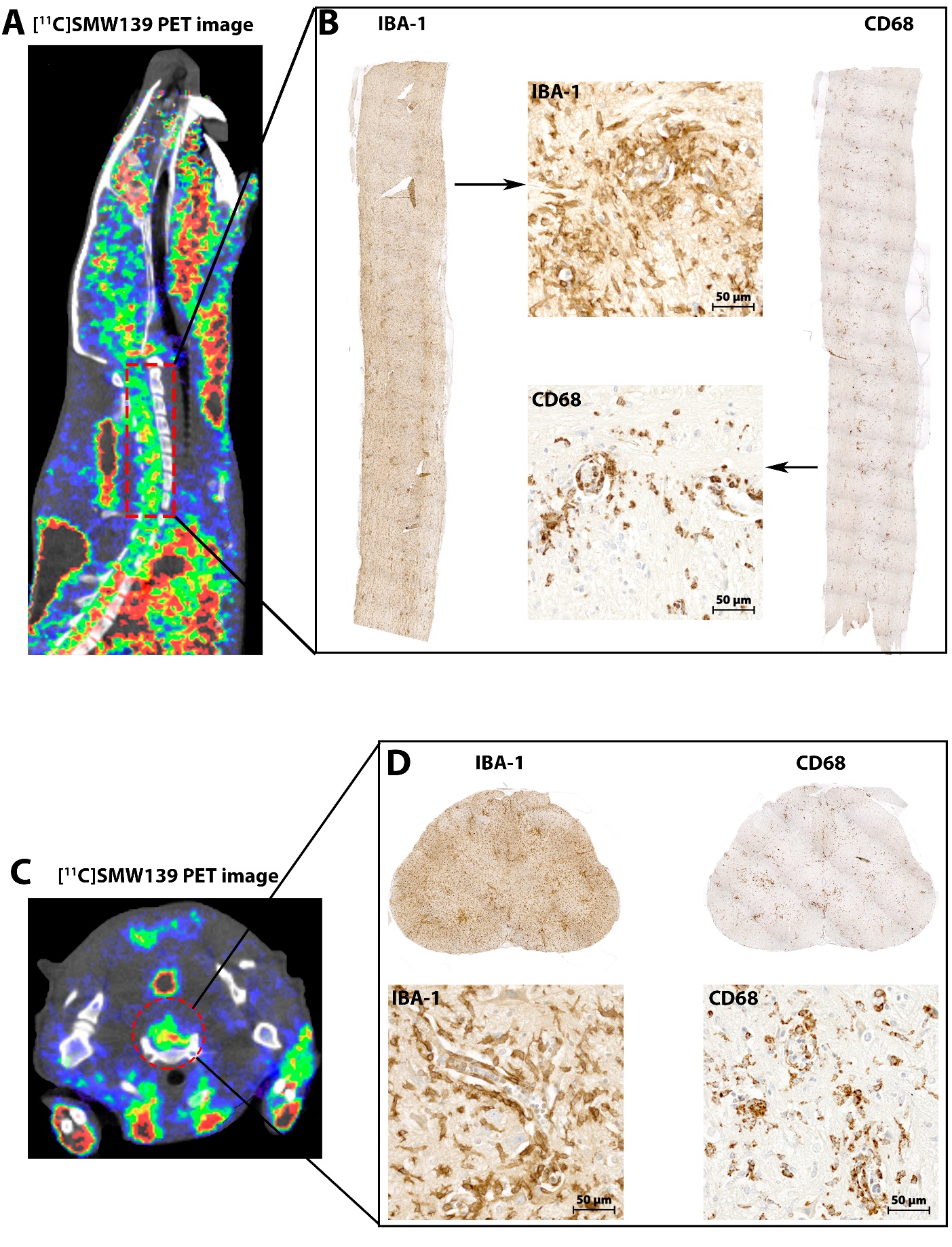


**Legend:** Correlation between uptake of [^11^C]SMW139 tracer in the spinal cord of the EAE animals and the ex vivo immunostaining for IBA-1 and ED-1.

**Supp. Figure 13.**

Correlation between uptake of [^11^C]SMW139 tracer in the spinal cord of the EAE animals and the ex vivo immunostaining for CD11b (microglia) and P2X_7_R; Sagittal (A) and transversal (C) PET image section showing the uptake of the [^11^C]SMW139 in the spinal cord. The dotted red circles or rectangles mark the area with the highest uptake. The green and red spots in the spinal cord indicate a high accumulation of [^11^C]SMW139 (A, C); Immunostaining with CD11b and P2X_7_R (B, D) of the respective brain region post PET imaging showing high microglia activation that expresses P2X_7_R in the same region where the high uptake of [^11^C]SMW139 was observed by PET imaging.


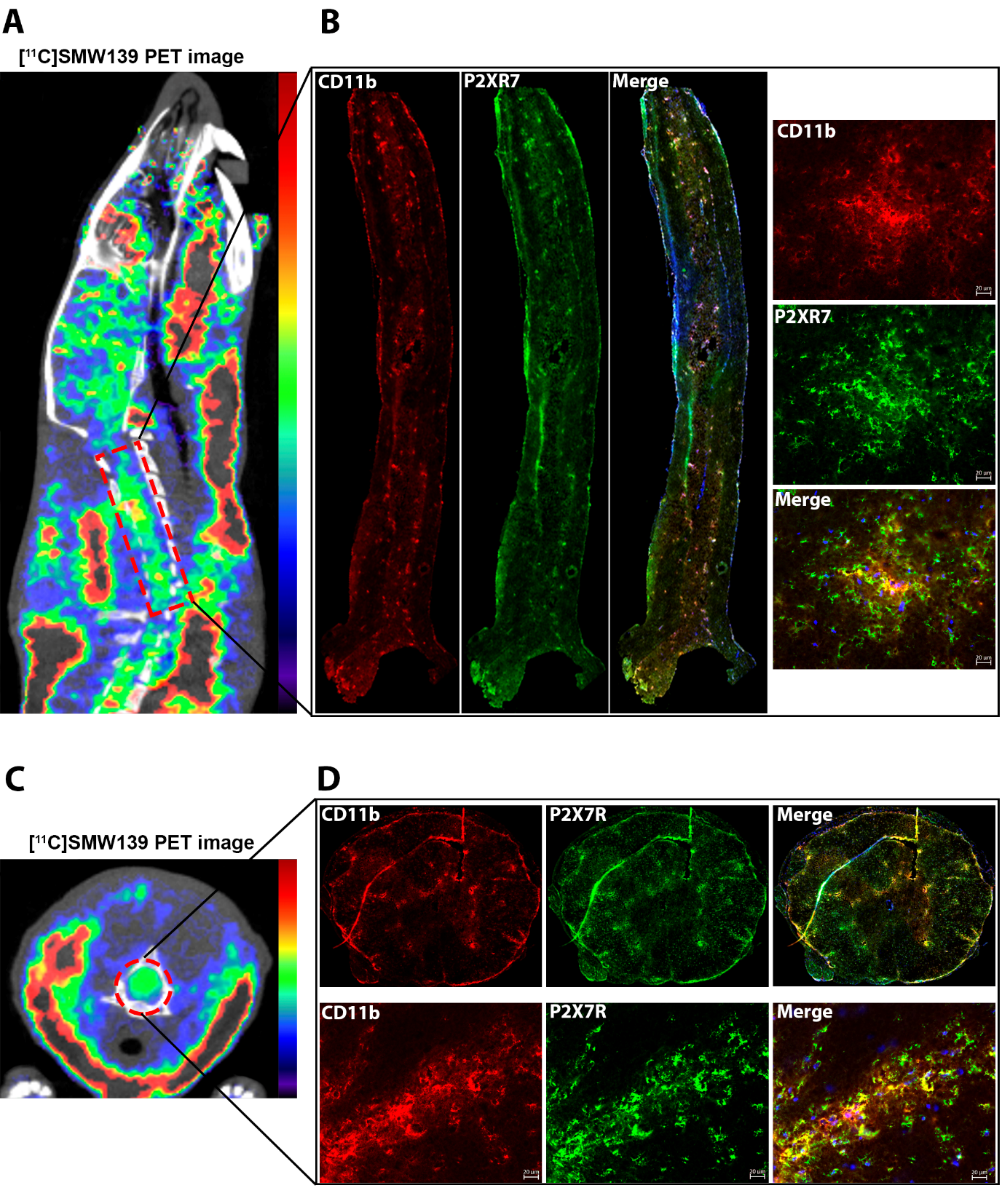
 **Legend:** Correlation between uptake of [^11^C]SMW139 tracer in the spinal cord of the EAE animals and the ex vivo immunostaining for CD11b (microglia) and P2X_7_R.

**Supp. Figure 14.**

Correlation between uptake of [^11^C]SMW139 tracer in the brain of the EAE animals and the ex vivo immunostaining for CD11b and P2X_7_R; Sagittal PET image section showing the uptake of the [^11^C]SMW139 in the brain. The area with the high uptake is marked by the doted red rectangle or circle. The green and red spots in the spinal cord indicate a high accumulation of [^11^C]SMW139 (A); Immunostaining with CD11b (microglia) and P2X_7_R (B, C) of the respective brain region post PET imaging showing high microglia activation in the same region where the high uptake of [^11^C]SMW139 was observed by PET imaging.


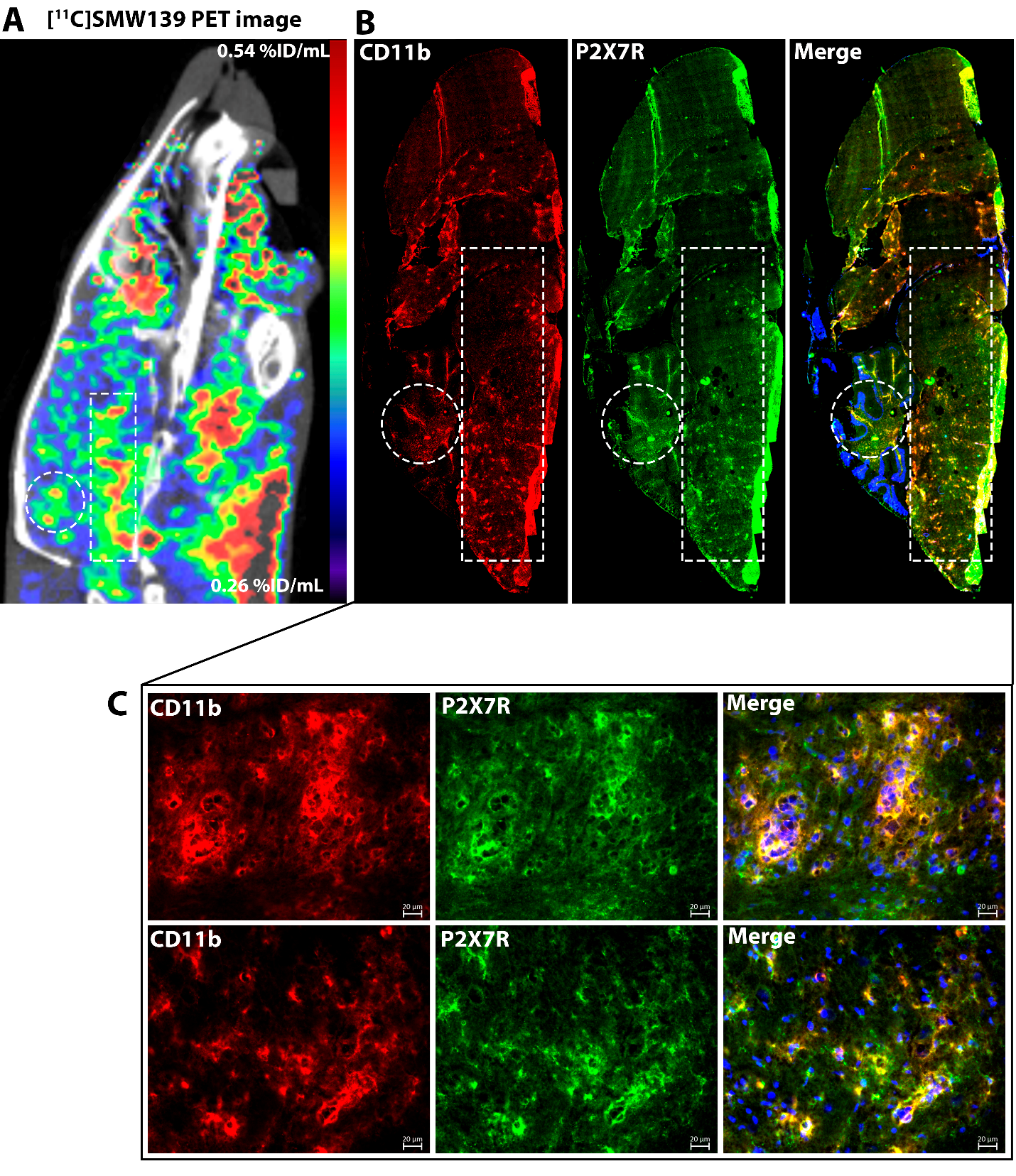


**Legend:** Correlation between uptake of [^11^C]SMW139 tracer in the brain of the EAE animals and the ex vivo immunostaining for CD11b and P2X_7_R.
